# Metformin use and risk of cancer in patients with type 2 diabetes: a cohort study of primary care records using inverse probability weighting of marginal structural models

**DOI:** 10.1093/ije/dyz005

**Published:** 2019-02-06

**Authors:** Ruth E Farmer, Deborah Ford, Rohini Mathur, Nish Chaturvedi, Rick Kaplan, Liam Smeeth, Krishnan Bhaskaran

**Affiliations:** 1 Department of Non Communicable Diseases Epidemiology, London School of Hygiene & Tropical Medicine, London, UK; 2 MRC Clinical Trials Unit at UCL, University College London, London, UK; 3 Institute for Cardiovascular Science, University College London, London, UK

**Keywords:** Marginal structural models, inverse probability weighting, type 2 diabetes, metformin, cancer, pharmacoepidemiology, time-dependent confounding

## Abstract

**Background:**

Previous studies provide conflicting evidence on whether metformin is protective against cancer. When studying time-varying exposure to metformin, covariates such as body mass index (BMI) and glycated haemoglobin (HbA1c) may act as both confounders and causal pathway variables, and so cannot be handled adequately by standard regression methods. Marginal structural models (MSMs) with inverse probability of treatment weights (IPTW) can correctly adjust for such confounders. Using this approach, the main objective of this study was to estimate the effect of metformin on cancer risk compared with risk in patients with T2DM taking no medication.

**Methods:**

Patients with incident type 2 diabetes (T2DM) were identified in the Clinical Practice Research Datalink (CPRD), a database of electronic health records derived from primary care in the UK. Patients entered the study at diabetes diagnosis or the first point after this when they had valid HbA1c and BMI measurements, and follow-up was split into 1-month intervals. Logistic regression was used to calculate IPTW; then the effect of metformin on all cancers (including and excluding non-melanoma skin cancer) and breast, prostate, lung, colorectal and pancreatic cancers was estimated in the weighted population.

**Results:**

A total of 55 629 T2DM patients were alive and cancer-free at their study entry; 2530 people had incident cancer during a median follow-up time of 2.9 years [interquartile range (IQR) 1.3–5.4 years]. Using the MSM approach, the hazard ratio (HR) for all cancers, comparing treatment with metformin with no glucose-lowering treatment, was 1.02 (0.88–1.18). Results were robust to a range of sensitivity analyses and remained consistent when estimating the treatment effect by length of exposure. We also found no evidence of a protective effect of metformin on individual cancer outcomes.

**Conclusions:**

We find no evidence that metformin has a causal association with cancer risk.

**Key Messages**

- Evidence for a protective effect of metformin on cancer risk remains under debate.
- Existing studies of metformin and cancer may not appropriately deal with time-dependent confounders affected by previous treatment.
- Inverse probability weighting of marginal structural models can deal with such confounders.
- Using this approach in a cohort of 55 000 patients with newly diagnosed diabetes from the Clinical Practice Research Datalink [median follow-up 2.9 years (IQR 1.3–5.4 years)] produced no evidence of an association between metformin use and cancer incidence.

## Introduction

Metformin is the preferred first-line treatment for type 2 diabetes (T2DM) in general practices in the UK.^1^^,^^2^ Previous epidemiological studies have suggested that metformin may reduce cancer incidence in patients with type 2 diabetes^3^^,^^4^; others have found no such association.^5–7^ The potential for bias in many studies has been highlighted previously.^8^^,^^9^

The highest-quality existing observational studies have compared new users of metformin with new users of sulphonylureas, ignoring subsequent changes in treatment (intention to treat approach),^9^ and finding no evidence of an association between metformin and cancer. However, the use of an active comparator makes it more difficult to attribute any observed effect (or lack thereof) to metformin itself. Further, patients inevitably switch treatment through time, so this approach may dilute any real association.

One possible randomized controlled trial (RCT) designed to examine the causal association between metformin use and cancer incidence might randomize patients with newly diagnosed type 2 diabetes to receive either metformin monotherapy or placebo, with all participants additionally advised to follow a standard diet and exercise regimen, and long follow-up to detect cancer outcomes. Provided there were no/minimal protocol deviations, notably that patients stayed on their allocated treatment through follow-up, such a trial would reliably estimate the effect of metformin on cancer risk. In reality, although practical constraints preclude such a trial, we may be able to recreate a similar comparison in routinely collected primary care records by comparing initiators and non-initiators of metformin through time. In the absence of randomization, there is likely to be time-dependent confounding by factors associated with both treatment initiation and cancer risk. In the presence of time-dependent confounders that are themselves likely to be affected by previous treatment, such as body mass index (BMI) and glycated haemoglobin (HbA1c),^10^^,^^11^ standard statistical methods are unable to estimate an unbiased treatment effect.^12^ Marginal structural models (MSMs) with inverse probability of treatment weighting (IPTW) are an established causal inference method to address such time-dependent confounding.^13^ The method creates a weighted population in which treatment initiation through time is independent of the time-dependent confounders, and has been widely used in the HIV literature to assess treatment regimens while controlling for time-varying CD4.^14^^,^^15^

To date, no studies have used MSMs with IPTW to compare cancer risk between new users of metformin, and patients with a diabetes diagnosis who are yet to initiate any treatment (no medication). Nor have they investigated the potential for time-dependent confounders when modelling time-varying treatment in the context of metformin and cancer. The main objective of this study was to estimate the causal effect of metformin monotherapy vs no medication on risk of cancer in patients with newly diagnosed T2DM, using MSMs with IPTW to appropriately deal with time-dependent confounding. We further aimed to evaluate the impact of adjusting for time-dependent confounders by comparing MSMs with standard methods.

## Methods

This study is based in part on data from the Clinical Practice Research Datalink obtained under licence from the UK Medicines and Healthcare products Regulatory Agency. The data are provided by patients and collected by the NHS as part of their care and support. The interpretation and conclusions contained in this study are those of the author/s alone. The study was approved by the Independent Scientific Advisory Committee (approval number: 12_027RA). The approved protocol was made available to the journal and reviewers during peer review. Generic ethical approval for observational research using the Clinical Practice Research Datalink (CPRD) with approval from ISAC has been granted by a Health Research Authority (HRA) Research Ethics Committee (East Midlands – Derby, REC reference number 05/MRE04/87). In addition, the study was approved by the London School of Hygiene and Tropical Medicine ethics committee (approval number 6349).

### Basic study population

Patients with incident T2DM were identified from the CPRD [https://www.cprd.com], using an algorithm developed previously^16^ (see Supplementary Methods, available as Supplementary data at *IJE* online). The algorithm required a diagnosis code for T2DM alongside either a diabetes-care related code or a prescription for an oral glucose-lowering medication [identified using British National Formulary (BNF) codes^17^]. Individuals became eligible when both codes required to fulfil the inclusion criteria were present, and this was taken as the date of diabetes diagnosis. If there were >30 days between the first and last code that confirmed the diagnoses, or <12 months observation preceding the first relevant code, the patient was excluded on the grounds that they might not be an incident case.

Patients with previous cancer, aged <30 or >90 at diagnosis, or with missing smoking/alcohol information at diabetes diagnosis, were excluded. A minority of patients lacked a valid BMI or HbA1c (measured within the previous 3 months) at diagnosis; for these individuals, study entry (baseline) was delayed until the point in follow-up when complete data were available.^18^ However, patients who had already commenced glucose-lowering therapy by this point were excluded (see Supplementary Methods, available as Supplementary data at *IJE* online).

Follow-up ended at the earliest of the following: death, leaving the practice, first cancer record, initiation of any glucose-lowering medication other than metformin, or the last data collection date from the practice (31 July 2014 at the latest).

### Exposure definition

The exposure of interest was metformin monotherapy, and the comparator group was patients with T2DM not taking any pharmacological therapy (hereafter referred to as ‘no medication’ controls). The date of metformin initiation was defined as the date of the first prescription record for metformin in CPRD (BNF code 6.1.2.2.2). The exposure status of individuals starting in the no medication group, who initiated metformin during follow-up, was time-updated in the month of the first metformin prescription. Patients were assumed to stay on metformin after their first prescription, since cessation of metformin without introduction of another glucose-lowering medication would be unusual and contrary to national guidance.^2^ All patients were censored at the initiation of any other glucose-lowering medication, in order to estimate an ‘as treated’ effect of metformin monotherapy.

### Outcomes

Cancer outcomes were identified using Read codes recorded in the patient’s CPRD record as described previously.^10^ The primary outcomes were all cancers combined, first including and then excluding non-melanoma skin cancer (NMSC). Breast, prostate, colorectal and lung cancer outcomes were investigated individually, as these were the four most common cancers; and pancreatic cancer was investigated as an outcome due to its known association with T2DM.^19^ The date of cancer diagnosis was brought forward by 6 months in the primary analysis to minimize reverse causality driven by undiagnosed cancer affecting diabetes control and thus treatment.

### Statistical analysis

**Figure 1.**
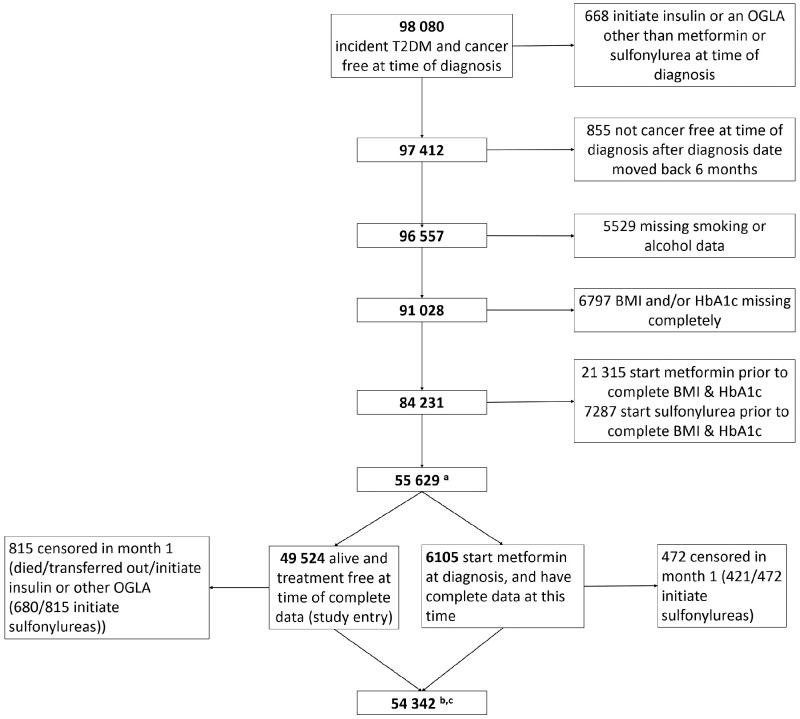
Flow chart to show how final analysis samples were obtained from 98 080 patients in CPRD with incident T2DM, who were cancer free at time of diabetes diagnosis; ^a^55 629 contribute to model for censoring weights; ^b^54 342 contribute to the outcome models; ^c^54 342 less those initiating metformin at baseline (6105) and 48 of the 49 524 treatment-naïve at study entry who had a cancer diagnosis in month 1 (not shown on figure) contributing to the model for the inverse probability of treatment weights (*n* = 48 661).

Inverse probability of treatment weighting (IPTW) of marginal structural models (MSMs) has been described elsewhere.^13^^,^^20^ Full details of the model fitting process for this analysis are given in Supplementary Material, available as Supplementary data at *IJE* online. Briefly, each patient’s data were expanded into monthly intervals, and pooled logistic regression models were fitted to estimate stabilized IPTW.^14^^,^^20^ Since we assumed patients remained exposed to metformin after their first prescription until the end of data collection or censoring, the probability of initiating metformin was estimated in each monthly interval up to and including the interval of metformin initiation. Patients who initiated metformin at diabetes diagnosis or who had the outcome in the first interval were not included in the weighting model, but contributed to the model for the effect of metformin on cancer (the ‘outcome model’) with a constant weight of one (see Supplementary Material, available as Supplementary data at *IJE* online; and Figure 1). The model for the denominator of the weight included time since study entry as the underlying time scale, modelled as a restricted cubic spline with knots at 0, 10, 25 and 120 months. Baseline covariates included in the model were: time between diabetes diagnosis and study entry (restricted cubic spline with knots at 0, 4 and 120 months); age in years (<45, 45–59, 60–75, >75); sex; calendar year (before 1995, 1995–99, 2000–04 and 2005 onwards); smoking (current, ex, never); alcohol consumption (non-drinker, ex-drinker, current drinker unknown quantity: rare drinker <2 units (u)/day (d), moderate drinker 3-6 u/d, excessive drinker >6 u/d); BMI (kg/m^2^) (<25, 25–29, 30–35 and >35); HbA1c (<6%, 6–6.5%, 6.5–7%, 7–8%, 8–10% and >10%); and indicator variables for: use of statins in the year preceding baseline; use of non-steroidal anti-inflammatory drugs (NSAIDs) in the year preceding baseline; use of anti-hypertensives in the year preceding baseline; history of cardiovascular disease (CVD); and history of chronic kidney disease (CKD). Time-varying covariates included: HbA1c in the previous month; BMI in the previous month; and indicator variables for: use of statins in the previous year, use of NSAIDs in the previous year, use of anti-HTs (anti-hypertensives) in the previous year; history of CVD; and history of CKD. Last observation carried forward (LOCF) was used to impute time-varying covariates going forward from study entry, if not measured in a particular interval. All baseline risk factors and time since study entry were entered into the model for the numerator of the weight.^14^^,^^20^ Stabilized inverse probability of censoring weights (IPCW) were calculated using a similar approach to account for non-informative censoring, and the distribution of the combined treatment and censoring weights was examined.^21^ Weights were truncated at 0.1 and 10.

The effect of metformin use on risk of cancer was estimated using pooled logistic regression models with time since study entry included in the model (as a cubic spline with the same knot points as the weighting model) to approximate a Cox proportional hazards model allowing for time-varying weights.^22^ Exposure to metformin was modelled using a binary variable to represent current treatment. For composite cancer endpoints, exposure was also modelled by time since first metformin prescription (assumed equivalent to cumulative time on metformin) categorized as no medication, 0–6 months, 6–12 months, 1–2 years, 2–5 years, 5–7 years and >7 years), as a time-varying exposure.

Four outcome models were fitted to evaluate the effect of metformin on cancer risk. The first three models were unweighted models with varying levels of confounder adjustment, namely: model 1—minimal adjustment (adjusted for baseline age, gender, smoking, alcohol and calendar year of diabetes onset); model 2—full baseline adjustment (as model 1 plus all other baseline covariates); model 3—adjustment for all baseline and time-dependent covariates.

The fourth model (the MSM) was a weighted model using the joint treatment and censoring weights. All baseline covariates included in the model for the numerator of the weights were included as covariates in the MSM. Both weighting and outcome models were repeated using restricted cubic splines for age, HbA1c and BMI, to investigate the impact of possible model mis-specification from inappropriate covariate form. All analysis was performed in Stata v.14.^23^ All code lists used in this study are available on the London School of Hygiene and Tropical Medicine (LSHTM) data compass.

### Sensitivity analyses

The three main sensitivity analyses were as follows. First, we varied how far forward the cancer diagnosis dates were brought (0 and 12 months), to assess the impact of different latency periods. Second, the interval length used was changed from 1 to 3 months. Third, we explored the effect of fitting treatment models separately by calendar period. More detail regarding the methods for these sensitivity analyses, and details of further (secondary) sensitivity analyses, are given in Supplementary Methods, available as Supplementary data at *IJE* online.

## Results

### Cohort description

A total of 98 080 patients with incident T2DM, aged 30–90 and with no history of any cancer at the time of diabetes diagnosis, were identified; 55 629 patients were eligible to enter the study (Table 1). The main reasons for non-inclusion were lack of HbA1c or BMI data before treatment initiation (Figure 1). The mean age at diabetes diagnosis was 62 years [standard deviation (SD) 12 years]. Median follow-up time was 2.9 years [interquartile range (IQR) 1.3 to 5.4 years], with 40% of included person-time on metformin.

**Table 1.** Demographics of included patients from the CPRD at study entry

	No medication *N* = 49 524	Metformin *N* = 6105	Total *N* = 55 629
Mean (SD) median, 25th percentile-75th percentile)			
Age at diagnosis (years)	62.2 (12) 63, 54-71	57.6 (11.8) 57, 49-66	61.7 (12) 62, 53 -71
HbA1c (%) at study entry	7.2 (1.6) 6.8, 6.2-7.7	9.4 (2.3) 9, 7.4-11	7.5 (1.8) 6.9, 6.3-8
BMI (kg/m^2^) at study entry	31.6 (6.3) 30.7, 27.3-34.9	33.4 (6.9) 32.3, 28.6-37.1	31.8 (6.3) 30.9, 27.5-35.2
*N* (%)			
Gender			
Male	27 763 (56.1)	3594 (58.9)	31 357 (56.4)
Female	21 761 (43.9)	2511 (41.1)	24 272 (43.6)
History of chronic kidney disease			
No	46 463 (93.8)	5866 (96.1)	52 329 (94.1)
Yes	3061 (6.2)	239 (3.9)	3300 (5.9)
History of cardiovascular disease			
No	41 868 (84.5)	5479 (89.7)	47 347 (85.1)
Yes	7656 (15.5)	626 (10.3)	8282 (14.9)
Use of statins in previous year			
No	25 035 (50.6)	2739 (44.9)	27 774 (49.9)
Yes	24 489 (49.4)	3366 (55.1)	27 855 (50.1)
Use of NSAID in previous year			
No	39 575 (79.9)	4999 (81.9)	44 574 (80.1)
Yes	9949 (20.1)	1106 (18.1)	11 055 (19.9)
Use of antihypertensive in previous year			
No	18 048 (36.4)	2767 (45.3)	20 815 (37.4)
Yes	31 476 (63.6)	3338 (54.7)	34 814 (62.6)
Smoking status			
Non	20 132 (40.7)	2449 (40.1)	22 581 (40.6)
Current	8746 (17.7)	1287 (21.1)	10 033 (18.0)
Ex	20 646 (41.7)	2369 (38.8)	23 015 (41.4)
Alcohol consumption			
Non-drinker	5770 (11.7)	884 (14.5)	6654 (12)
Ex-drinker	3474 (7)	529 (8.7)	4003 (7.2)
Current drinker quantity unknown	979 (2)	121 (2.0)	1100 (2.0)
Rare drinker <2 u/d	11 543 (23.3)	1484 (24.3)	13 027 (23.4)
Moderate drinker 3-6 u/d	22 934 (46.3)	2570 (42.1)	25 504 (45.8)
Excessive drinker >6 u/d	4824 (9.7)	517 (8.5)	5341 (9.6)
Calendar year of onset			
1990-95	134 (0.3)	0 (0)	134 (0.2)
1995-2000	1708 (3.5)	20 (0.3)	1728 (3.1)
2000-05	12 764 (25.8)	595 (9.8)	13 359 (24)
After 2005	34 918 (70.5)	5490 (89.9)	40 408 (72.6)

NSAID, non-steroidal anti-inflammatory drug.

During follow-up 2530 cancers were observed, with crude event rates for no medication and for metformin of 11.4 per 10 000 person-years and 9.4 per 10 000 person-years. respectively. There were 266 prostate, 241 breast, 185 lung, 226 colorectal and 50 pancreatic cancers observed during follow-up. The full breakdown of incident cancer types as defined by ICD-10 codes is given in Supplementary Table 1, available as Supplementary data at *IJE* online.

### Inverse probability weight estimation

The individuals who were more likely to initiate metformin had higher HbA1c, higher BMI, were of younger age and had a later calendar year of diagnosis. Model outputs for the estimation of both treatment and censoring weights are presented in Supplementary Tables 2–5, available as Supplementary data at *IJE* online. Following truncation, the mean of the weights was 1.00 (SD 1.09), with 1st and 99th percentiles of 0.1 and 6.48, respectively (Supplementary Tables 6 and 7, available as Supplementary data at *IJE* online).

Table 2 presents estimates of the hazard ratios (HRs) for the effect of metformin vs no medication on cancer risk in patients with newly diagnosed T2DM. For all cancer types examined except colorectal cancer, use of the MSM increased the HR compared with standard statistical methods that could not appropriately account for time-dependent confounders affected by previous treatment. For all cancers combined, this change was relatively small in magnitude, and all models were generally consistent with no effect of metformin on risk of cancer. For specific cancers, the changes between the unweighted and weighted models were more noticeable, though confidence intervals were wide. For pancreatic cancer, all models estimated an increased risk with metformin use, with the highest excess risk estimated by the MSM [HR 3.11, 95% confidence interval (CI) 1.24, 7.76]. For colorectal cancer, the MSM estimated a reduction [HR 0.71, 95% CI 0.43, 1.18] in risk of cancer in patients using metformin vs no medication.

**Table 2.** Hazard ratio, 95% CI and *P*-value for the effect of metformin vs no medication on risk of cancer in patients with newly diagnosed diabetes, from four models with varying level of covariate adjustment

	All cancers (inc. NMSC) (2530 events)		All cancers (excl. NMSC) (2000 events)		Breast cancer (241 events)		Prostate cancer (266 events)		Lung cancer (185 events)		Pancreatic cancer (50 events)		Colorectal cancer (226 events)	
	HR	95% confidence interval	HR	95% confidence interval	HR	95% confidence interval	HR	95% confidence interval	HR	95% confidence interval	HR	95% confidence interval	HR	95% confidence interval
Model 1: basic baseline adjustment	0.91	(0.84, 1.00)	0.94	(0.85, 1.04)	0.82	(0.62, 1.07)	1.05	(0.8, 1.38)	0.93	(0.68, 1.28)	2.25	(1.26, 4.04)	0.95	(0.72, 1.26)
Model 2: full baseline adjustment	0.95	(0.86, 1.04)	0.94	(0.84, 1.06)	0.82	(0.61, 1.11)	1.08	(0.8, 1.47)	0.99	(0.71, 1.37)	1.96	(0.96, 4.03)	0.88	(0.64, 1.21)
Model 3: baseline and time updated adjustment	0.94	(0.85, 1.03)	0.94	(0.84, 1.05)	0.86	(0.63, 1.17)	1.10	(0.81, 1.51)	1.01	(0.72, 1.40)	1.66	(0.85, 3.24)	0.82	(0.59, 1.13)
Model 4: MSM with IPTW and IPCW	1.02	(0.88, 1.18)	1.05	(0.89, 1.25)	0.94	(0.62, 1.43)	1.09	(0.72, 1.65)	1.26	(0.77, 2.06)	3.11	(1.24, 7.76)	0.71	(0.43, 1.18)

Estimates from three standard analysis methods (1–3) and one MSM with joint IPTW and IPCW (4).

Model 1: Minimal adjustment for confounding: adjustment for age, gender, smoking status and alcohol status and year of onset of diabetes.

Model 2: Full adjustment for baseline covariates: model 1 + baseline adjustment for: HbA1c, BMI, use of other medications in previous year (NSAIDS, statins, antihypertensive drugs), history of chronic kidney disease (CKD) and cardiovascular disease (CVD).

Model 3: Full adjustment for baseline covariates with time-dependent covariates added: model 2 + adjustment for time-updated HbA1c, BMI, and history of CVD, CKD and use of other medications in the past 12 months.

Model 4: As Model 2, weighted using joint IPTW and IPCW (MSM with IPTW and IPCW). HRs approximated from a pooled logistic regression.

Inc., including; excl., excluding.

**Figure 2.**
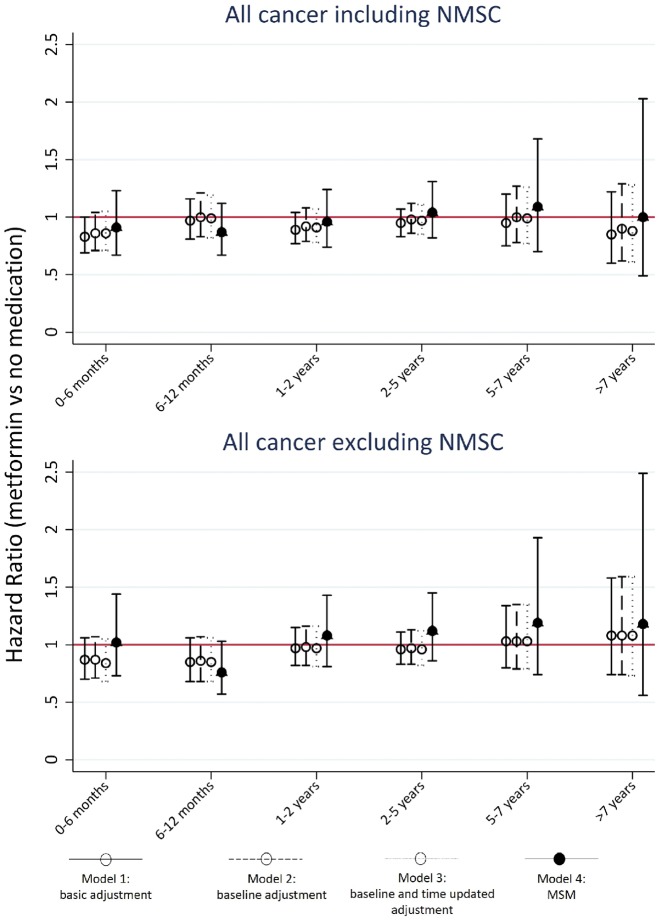
Hazard ratios and 95% confidence intervals for effect of metformin use on risk of cancer, estimated by time since first metformin prescription. Top: All cancers including NMSC. Bottom: All cancers excluding NMSC. Estimates from three standard analysis methods (1–3) and MSM with joint IPTW and IPCW (4). Model 1: Minimal adjustment for confounding: adjustment for age, gender, smoking status and alcohol status and year of onset of diabetes. Model 2: Full adjustment for baseline covariates: model 1 + baseline adjustment for: HbA1c, BMI, use of other medications in previous year (NSAIDS, statins, antihypertensive drugs), history of chronic kidney disease (CKD) and cardiovascular disease (CVD). Model 3: Full adjustment for baseline covariates with time-dependent covariates added: model 2 + adjustment for time-updated HbA1c, BMI, and history of CVD, CKD and use of other medications in the past 12 months. Model 4: As model 2, weighted using joint IPTW and IPCW (MSM with IPTW and IPCW). HRs approximated from a pooled logistic regression. NMSC, non melanoma skin cancer.

When stratifying by cumulative exposure to metformin, the MSM gave results consistent with no effect of metformin on risk of all cancers combined for all time periods, though precision of the estimates reduced as the length of exposure increased, due to loss of power (Figure 2). The unweighted models had similar results, though for all cancer excluding NMSC these models tended to estimate a lower risk with metformin use for most time periods, albeit with confidence intervals that overlapped those from the weighted analysis (Figure 2; Supplementary Tables 8 and 9, available as Supplementary data at *IJE* online).

### Sensitivity analyses

**Figure 3.**
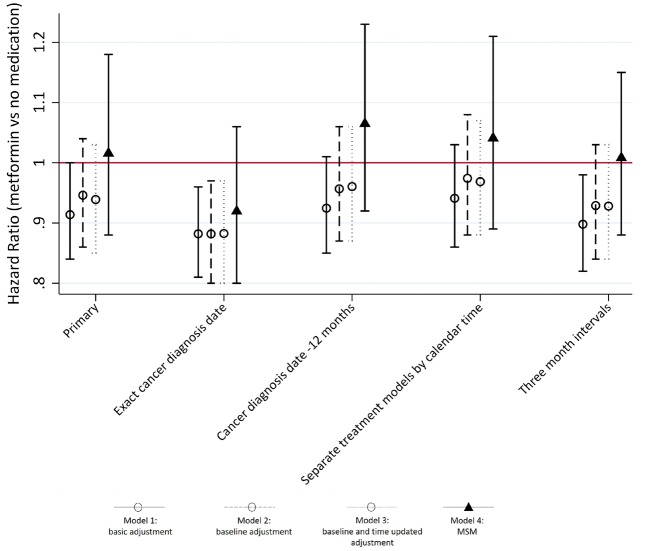
Hazard ratios and 95% confidence intervals for effect of metformin use on risk of cancer for primary analysis (left) and 4 sensitivity analyses. Estimates from three standard analysis methods (1–3) and MSM with joint IPTW and IPCW (4). Model 1 – Minimal adjustment for confounding: adjustment for age, gender, smoking status and alcohol status and year of onset of diabetes. Model 2 Full adjustment for baseline covariates: Model 1 + baseline adjustment for: HbA1c, BMI, use of other medications in previous year (NSAIDS, statins, antihypertensive drugs), history of chronic kidney disease (CKD) and cardiovascular disease (CVD). Model 3 – Full adjustment for baseline covariates with time-dependent covariates added: Models 2 + adjustment for time updated HbA1c, BMI, and history of CVD, CKD and use of other medications in the past 12 months. Model 4 – As Model 2, weighted using joint IPTW and IPCW (MSM with IPTW and IPCW). HRs approximated from a pooled logistic regression.

None of the sensitivity analyses produced meaningfully different results to those observed in the primary analysis (see Figure 3; and Supplementary Figure 1, available as Supplementary data at *IJE* online). Outcome models using cubic spline parameterizations of continuous covariates gave similar results (Supplementary Table 10, available as Supplementary data at *IJE* online).

## Discussion

We found no evidence of association between metformin use and overall risk of cancer in patients with T2DM. This finding was consistent across a range of sensitivity analyses, and in analyses stratified by cumulative exposure to metformin. We also found no evidence of association between metformin and breast, colorectal or lung cancer, though precision was lower for these outcomes. We observed an increased risk of pancreatic cancer.

The majority of well-designed previous studies have compared new users of metformin with new users of an alternative first-line diabetes therapy such as a sulphonylurea, with covariate adjustment at the time of first exposure.^9^ Although answering a slightly different question, the results of these studies are generally consistent with the analyses presented here even where causal models to account for time-dependent confounding were not used.^5^^,^^6^^,^^24^ One study, using causal methodology to account for informative loss to follow-up, compared cancer risk between new users of metformin and new users of sulphonylureas.^6^ Though a different comparison group was used, results were consistent with our findings: the authors found no difference in risk of any cancer [HR for metformin vs sulfonylureas 0.94, 95% CI 0.85, 1.04]. Findings for individual cancers were broadly consistent with our individual cancer estimates, except for a suggestion of a protective effect of metformin on pancreatic cancer, in contrast with the increased risk of pancreatic cancer with metformin use vs no medication in the present study. An early symptom of pancreatic cancer may be onset of type 2 diabetes, and it is possible that the increased risk we observed was driven by undiagnosed cancer causing more severe onset and/or an indication for metformin that is not captured by our weighting models. Although we attempted to remove such reverse causality using a 6-month lag time, this may have been insufficient for pancreatic cancer, which is often diagnosed late.

For most outcomes the MSMs produced results that were similar to those obtained via standard analysis methods with baseline adjustment only, despite the hypothesized presence of time-dependent confounders affected by previous treatment. This may suggest that the time-dependent confounding was not as strong as initially thought, and as such, that previous well-designed studies would be unlikely to find contrasting results with a marginal structural model approach. Hicks (2017)^25^ found a similar lack of difference in estimates between standard analysis methods and MSMs, when comparing metformin with any other oral glucose-lowering drug for risk of virally associated cancers. In that study, 86% of individuals prescribed metformin during follow-up were using it at the time of cohort entry, meaning that only a small proportion of individuals would have been affected by time-dependent confounding. In our analysis, there were many more patients unexposed to metformin at study entry, but the overall median time to initiation was only 2 months (IQR 1–16 months). Therefore it is possible that not enough patients were initiating treatment far enough after baseline for the time-varying confounders to change sufficiently. In the analysis of cumulative medication, the differences in estimates for more than 7 years’ exposure between standard methods and MSM were greater, which is consistent with this explanation.

It is also possible that in combining all cancers, any potential time-dependent confounding was masked because the confounding acts in opposite directions for different cancers. In particular, the association between BMI and risk of cancer has been shown to differ by cancer type.^10^^,^^26^ The slightly larger observed changes between standard models and MSMs in some of the site-specific analyses support this possibility, though the site-specific analyses did not produce results that suggested that a protective effect of metformin was being masked by combining cancers into a composite endpoint.

An important limitation of this analysis is that the average follow-up time of patients in this study was relatively short. With a median time of 2.9 years, we acknowledge that there may have been insufficient follow-up in enough patients to detect any causal effect of metformin on cancer. The decision to censor at initiation of any other therapy in our analyses contributed to this reduced follow-up time, but with use of IPCW to adjust for informative censoring, this approach was deemed the most appropriate way to remove issues of treatment switching. It should also be noted that previous studies of metformin and cancer with contrasting results also had average follow-up times that are broadly comparable to our study.^3^^,^^4^ By stratifying by length of exposure to metformin in a secondary analysis, it was possible to obtain an estimate for the effect of 5–7 and more than 7 years of metformin use on cancer risk, and the point estimates remained close to the null. However, we must acknowledge that due to fewer numbers with long-term follow-up, confidence intervals for more than 7 years of exposure cannot rule out up to a 51% decreased risk or 103% increased risk for all cancers combined. Additionally, there may have been residual confounding by physical activity and diet, data for which are not available in the CPRD, or by smoking, for which only crude data were available.

Previous studies have found that cancer diagnoses taken from CPRD primary care data have good concordance with external sources, and have a low false-positive rate.^27^^,^^28^ However, feedback of cancer diagnoses from secondary care to GPs may be imperfect, and we cannot exclude the possibility of some under-ascertainment of our outcomes by relying on primary care data alone. The effect of any under-ascertainment of outcomes on the estimated effects of metformin is likely to be small, since the hazard ratio remains unbiased when the misclassification affects sensitivity but not specificity.^29^

In our analysis, only a single prescription was required to be considered exposed to metformin, and it was assumed that the patient remained exposed until there was evidence of a change in medication; however, this approach would not take into account non-adherence to prescribed medication or cessation of all antidiabetic therapy. It should also be acknowledged that patients apparently off treatment may have been receiving medication from specialist diabetes clinics or other sources. However, since diabetes is predominantly managed in primary care,^30^ this is unlikely to affect the results substantially. Indeed, a strength is that the patients captured by our study, namely a cohort of newly diagnosed type 2 diabetes patients receiving primary care from their general practitioner, is a highly relevant population for this question—especially since metformin and lifestyle changes are both common first-line interventions in clinical practice.^2^

Even after weight stabilization, there were extremely large weights for some individuals, usually driven by the characteristic of having a high HbA1c but not initiating metformin. Truncation of the stabilized weights was therefore necessary, which may have resulted in re-introducing time-dependent confounding. However, we only truncated the top 0.6% of the weights, meaning that under the assumption that the weighting model was correctly specified, the amount of confounding re-introduced is likely to be small.

We found that many patients had missing HbA1c or BMI at the time of diabetes diagnosis. Use of multiple imputation was not considered since there is limited research on the use of multiple imputation with MSMs, and it is likely impractical to combine these methods in a large dataset, due to computational intensity. To increase numbers, patients entered the study at the first point (at or after diabetes diagnosis) at which they had data on all covariates, as long as this was before any treatment was initiated. Using this approach instead of a complete case analysis increased the sample size by about 20 000 patients, and since 75% of these patients entered the study within 6 months of their diagnosis date, it was considered that this would not cause serious bias. However, this approach resulted in the exclusion of a large number of individuals who initiated treatment before they obtained measurements for HbA1c and BMI. This could induce selection bias if the reason for not having measurements was related to cancer risk, though there is no clear reason why this would be true.

We believe this to be the first published study to assess cancer risk associated with metformin use vs no medication while appropriately adjusting for time-dependent confounders affected by previous treatment. We found no evidence that metformin has a protective effect on cancer risk in patients with type 2 diabetes—a result consistent with some existing studies using new-user active comparator designs and an intention to treat approach. Although we acknowledge loss of precision, our results had consistent estimates close to the null when looking by length of exposure. As such, these results add weight to the view that the large protective effects previously observed were not causal.

## Funding

This study was funded by a Medical Research Council doctoral studentship award to R.F. K.B. holds a Sir Henry Dale Fellowship jointly funded by the Wellcome Trust and the Royal Society (107731/Z/15/Z). R.M. is supported by a Wellcome Postdoctoral Fellowship from the Wellcome Trust (201375/Z/16/Z). D.F. and R.K. are supported by core support to the Medical Research Council (MC-UU-12023/20; MC-UU-12023/21).

**Conflict of interest:** The authors declare no conflicts of interest.

## Supplementary Material

Supplementary Data
