# Supplementary material for: Metformin use and risk of cancer in patients with type 2 diabetes: a cohort study of primary care records using inverse probability weighting of marginal structural models
Source: Int J Epidemiol. 2019 Feb 6;48(2):527–37. doi: 10.1093/ije/dyz005 (PMC6469299; doi:10.1093/ije/dyz005)
Supplement: Supplementary Data [file dyz005_supp.zip › dyz005-Suppl_data/dyz005_Supp_1.docx]

| ICD code/Site | Number of events | % |
| --- | --- | --- |
| C43 & 44 /Malignant Skin Cancer | 738 | 29 |
| C61/Prostate Cancer | 266 | 11 |
| C50/Breast Cancer | 241 | 10 |
| C34/Lung Cancer | 185 | 7 |
| C18/Colon Cancer | 158 | 6 |
| C67/Bladder Cancer | 69 | 3 |
| C25/Pancreatic Cancer | 50 | 2 |
| C15/Oesophageal Cancer | 65 | 3 |
| C20/Rectal Cancer | 68 | 3 |
| C54/Endometrial Cancer | 47 | 2 |
| C85/ Lymphoma | 40 | 2 |
| Other* | 603 | 24 |
| Total | **2530** | **100** |

Supplementary table 1: Frequency table of cancer types occurring in study cohort

*all other cancers with less than 40 events each, plus ICD C80 for “cancer of unspecified site”

|  | Stabilised | truncated at 1st and 99th %iles | truncated at 0.1 and 10 |
| --- | --- | --- | --- |
| Mean | 4.3 | 0.97 | 1.00 |
| SD | 832.5 | 0.86 | 1.06 |
| 1st %ile | 0.05 | 0.05 | 0.10 |
| 5th %ile | 0.16 | 0.16 | 0.16 |
| 50th %ile | 0.86 | 0.86 | 0.86 |
| 95th %ile | 2.25 | 2.25 | 2.25 |
| 99th %ile | 6.48 | 6.48 | 6.48 |
| Minimum | 0.00001 | 0.05 | 0.10 |
| Maximum | 608003 | 6.48 | 10 |

Supplementary table 6: Distribution of joint inverse probability of treatment and inverse probability of censoring weights (stabilised and two different truncations).

|  | Stabilised | truncated at 1st and 99th %iles | truncated at 0.1 and 10 |
| --- | --- | --- | --- |
| Mean | 150885 | 0.97 | 1.00 |
| SD | 79500000 | 0.88 | 1.07 |
| 1st %ile | 0.04 | 0.04 | 0.10 |
| 5th %ile | 0.15 | 0.15 | 0.15 |
| 10th %ile | 0.30 | 0.30 | 0.30 |
| 25th %ile | 0.65 | 0.65 | 0.65 |
| 50th %ile | 0.88 | 0.88 | 0.88 |
| 75th %ile | 0.98 | 0.98 | 0.98 |
| 90th %ile | 1.35 | 1.35 | 1.35 |
| 95th %ile | 2.14 | 2.14 | 2.14 |
| 99th %ile | 6.83 | 6.83 | 6.83 |
| Minimum | 0.00 | 0.04 | 0.10 |
| Maximum | 7.57x10^10^ | 6.83 | 10 |

Supplementary table 7: Distribution of joint inverse probability of treatment and inverse probability of censoring weights ( stabilised and two different truncations) from treatment models with more complex covariate specifications (cubic splines for continuous covariates).

|  | DENOMINATOR MODEL | | | NUMERATOR MODEL | | |
| --- | --- | --- | --- | --- | --- | --- |
|  | **OR** | **SE** | **95% CI (OR)** | **OR** | **SE** | **95% CI (OR)** |
| BASELINE FIXED |  |  |  |  |  |  |
| *Time since study entry (months) spl 1 | 0.98 | 0.001 | 0.98 , 0.98 | 0.98 | 0.001 | 0.98 , 0.98 |
| *Time since study entry (months) spl 2 | 1.05 | 0.004 | 1.04 , 1.06 | 1.08 | 0.004 | 1.07 , 1.08 |
| *Time between diagnosis and study entry spl 1 | 0.87 | 0.022 | 0.82 , 0.91 | 0.85 | 0.027 | 0.80 , 0.91 |
| *Time between diagnosis and study entry spl 2 | 1.06 | 0.020 | 1.02 , 1.10 | 0.86 | 0.020 | 0.82 , 0.90 |
| Age at diagnosis (years) |  |  |  |  |  |  |
| 32-44 | 1 (ref) |  |  | 1 (ref) |  |  |
| 45-59 | 0.97 | 0.032 | 0.91 , 1.04 | 0.96 | 0.330 | 0.90 , 1.02 |
| 60-74 | 0.88 | 0.029 | 0.83 , 0.94 | 0.76 | 0.024 | 0.71 , 0.81 |
| 75-89 | 0.63 | 0.025 | 0.58 , 0.68 | 0.51 | 0.019 | 0.47 , 0.55 |
| Gender (F v M) | 1.13 | 0.019 | 1.09 , 1.17 | 1.12 | 0.019 | 1.08 , 1.15 |
| Smoking Status |  |  |  |  |  |  |
| Non | 1 (ref) |  |  |  |  |  |
| Current | 1.03 | 0.024 | 0.99 , 1.08 | 1.10 | 0.025 | 1.06 , 1.15 |
| Ex | 1.07 | 0.019 | 1.03 , 1.10 | 1.07 | 0.019 | 1.04 , 1.11 |
| Alcohol consumption |  |  |  |  |  |  |
| non_drinker | 1 (ref) |  |  |  |  |  |
| ex-drinker | 0.97 | 0.040 | 0.90 , 1.06 | 0.96 | 0.037 | 0.89 , 1.04 |
| current drinker unknown | 0.82 | 0.057 | 0.71 , 0.94 | 0.87 | 0.053 | 0.77 , 0.98 |
| rare drinker <2u/d | 0.98 | 0.028 | 0.93 , 1.04 | 0.97 | 0.027 | 0.92 , 1.03 |
| moderate drinker 3-6u/d | 0.98 | 0.026 | 0.93 , 1.03 | 0.94 | 0.024 | 0.89 , 0.99 |
| excessive drinker >6u/d | 0.93 | 0.033 | 0.86 , 0.99 | 0.87 | 0.030 | 0.81 , 0.93 |
| Year of diabetes onset |  |  |  |  |  |  |
| 1990-1994 | 1 (ref) |  |  |  |  |  |
| 1995-2000 | 1.30 | 0.268 | 0.87 , 1.95 | 1.19 | 0.243 | 0.79 , 1.77 |
| 2001-2005 | 1.33 | 0.273 | 0.89 , 1.99 | 1.14 | 0.229 | 0.77 , 1.69 |
| 2005 onwards | 1.39 | 0.286 | 0.93 , 2.08 | 1.11 | 0.224 | 0.75 , 1.65 |
| Use of anti HT in year prior to study entry | 0.87 | 0.027 | 0.81 , 0.92 | 0.97 | 0.017 | 0.93 , 1.00 |
| Use of statin in year prior to study entry | 0.89 | 0.021 | 0.85 , 0.93 | 1.21 | 0.021 | 1.17 , 1.25 |
| Use of NSAID in year prior to study entry | 1.09 | 0.025 | 1.04 , 1.14 | 1.16 | 0.021 | 1.12 , 1.20 |
| HbA1c at study entry |  |  |  |  |  |  |
| <6% | 1 (ref) |  |  |  |  |  |
| 6% - 6.5% | 0.86 | 0.031 | 0.80 , 0.92 | 1.61 | 0.045 | 1.52 , 1.70 |
| 6.5%-7% | 0.76 | 0.028 | 0.71 , 0.82 | 2.52 | 0.068 | 2.39 , 2.66 |
| 7% - 8% | 0.62 | 0.024 | 0.57 , 0.67 | 5.21 | 0.139 | 4.95 , 5.49 |
| 8%-10% | 0.44 | 0.020 | 0.40 , 0.48 | 10.26 | 0.329 | 9.64 , 10.93 |
| >10% | 0.42 | 0.025 | 0.38 , 0.48 | 15.9 | 0.670 | 14.64 , 17.27 |
| BMI at study entry |  |  |  |  |  |  |
| <25 | 1 (ref) |  |  |  |  |  |
| 25-29 | 0.96 | 0.046 | 0.88 , 1.06 | 1.41 | 0.043 | 1.33 , 1.49 |
| 30-34 | 0.89 | 0.051 | 0.79 , 0.99 | 1.55 | 0.048 | 1.46 , 1.65 |
| 35+ | 0.76 | 0.052 | 0.66 , 0.87 | 1.76 | 0.057 | 1.65 , 1.87 |
| History of CVD at study entry | 0.92 | 0.053 | 0.82 , 1.03 | 1.03 | 0.023 | 0.99 , 1.08 |
| History of CKD at study entry | 0.82 | 0.047 | 0.74 , 0.92 | 0.69 | 0.028 | 0.64 , 0.75 |
| TIME UPDATED |  |  |  |  |  |  |
| Use of anti HT in previous year | 1.07 | 0.024 | 1.03 , 1.12 |  |  |  |
| Use of statin in previous year | 1.55 | 0.037 | 1.48 , 1.63 |  |  |  |
| Use of NSAID in previous year | 1.21 | 0.039 | 1.14 , 1.29 |  |  |  |
| History of CVD | 1.03 | 0.057 | 0.93 , 1.15 |  |  |  |
| History of CKD | 0.87 | 0.036 | 0.80 , 0.94 |  |  |  |
| HbA1c in previous interval |  |  |  |  |  |  |
| <6% | 1 (ref) |  |  |  |  |  |
| 6% - 6.5% | 2.35 | 0.141 | 2.09 , 2.64 |  |  |  |
| 6.5%-7% | 7.13 | 0.409 | 6.37 , 7.98 |  |  |  |
| 7% - 8% | 41.58 | 2.357 | 37.21 , 46.47 |  |  |  |
| 8%-10% | 186.22 | 11.370 | 165.22 , 209.89 |  |  |  |
| >10% | 311.57 | 22.934 | 269.71 , 359.92 |  |  |  |
| Bmi in previous interval |  |  |  |  |  |  |
| <25 | 1 (ref) |  |  |  |  |  |
| 25-29 | 1.38 | 0.064 | 1.26 , 1.52 |  |  |  |
| 30-34 | 1.67 | 0.093 | 1.50 , 1.86 |  |  |  |
| 35+ | 2.21 | 0.147 | 1.94 , 2.52 |  |  |  |

Supplementary table 2: denominator and numerator model ouput for inverse probability of treatment weights using categorical covariate specifications (primary analysis).

|  | DENOMINATOR MODEL | | | NUMERATOR MODEL | | |
| --- | --- | --- | --- | --- | --- | --- |
|  | OR | SE | 95% CI | OR | SE | 95% CI |
| BASELINE FIXED COVARIATES |  |  |  |  |  |  |
| Time (months) since study entry |  |  |  |  |  |  |
| Time since study entry (months) spl 1 | 0.5 | 0.01 | 0.48 , 0.53 | 0.4 | 0.01 | 0.38 , 0.41 |
| Time since study entry (months) spl 2 | 1.61 | 0.04 | 1.53 , 1.69 | 2.36 | 0.06 | 2.25 , 2.47 |
| Time since study entry (months) spl 3 | 0.62 | 0.02 | 0.59 , 0.65 | 0.34 | 0.01 | 0.33 , 0.36 |
| Time since study entry (months) spl 4 | 1.2 | 0.02 | 1.17 , 1.24 | 1.48 | 0.02 | 1.44 , 1.52 |
| Time (months) between diagnosis and study entry |  |  |  |  |  |  |
| Time between diagnosis and study entry spl 1 | 0.87 | 0.02 | 0.82 , 0.92 | 0.85 | 0.03 | 0.8 , 0.91 |
| Time between diagnosis and study entry spl 2 | 1.07 | 0.02 | 1.03 , 1.12 | 0.85 | 0.02 | 0.81 , 0.89 |
| Age at diagnosis (years) |  |  |  |  |  |  |
| Age at diagnosis spl1 | 0.89 | 0.01 | 0.87 , 0.91 | 0.8 | 0.01 | 0.79 , 0.82 |
| Age at diagnosis spl2 | 0.92 | 0.01 | 0.9 , 0.94 | 0.91 | 0.01 | 0.9 , 0.93 |
| Age at diagnosis spl3 | 1.04 | 0.01 | 1.02 , 1.05 | 1.01 | 0.01 | 0.99 , 1.03 |
| Gender (FvM( | 1.15 | 0.02 | 1.11 , 1.19 | 1.12 | 0.02 | 1.08 , 1.16 |
| Smoking Status |  |  |  |  |  |  |
| Non | 1 (ref) |  |  | 1 (ref) |  |  |
| Current | 1.03 | 0.03 | 0.98 , 1.08 | 1.09 | 0.02 | 1.04 , 1.13 |
| Ex | 1.07 | 0.02 | 1.03 , 1.11 | 1.07 | 0.02 | 1.03 , 1.1 |
| Alcohol consumption |  |  |  |  |  |  |
| non_drinker | 1 (ref) |  |  | 1 (ref) |  |  |
| ex-drinker | 0.94 | 0.04 | 0.86 , 1.02 | 0.95 | 0.04 | 0.88 , 1.02 |
| current drinker unknown | 0.80 | 0.06 | 0.69 , 0.93 | 0.87 | 0.05 | 0.77 , 0.98 |
| rare drinker <2u/d | 0.96 | 0.03 | 0.91 , 1.02 | 0.97 | 0.03 | 0.92 , 1.02 |
| moderate drinker 3-6u/d | 0.96 | 0.03 | 0.91 , 1.02 | 0.92 | 0.02 | 0.88 , 0.97 |
| excessive drinker >6u/d | 0.92 | 0.03 | 0.86 , 0.99 | 0.86 | 0.03 | 0.8 , 0.92 |
| Year of diabetes onset |  |  |  |  |  |  |
| 1990-1994 | 1 (ref) |  |  | 1 (ref) |  |  |
| 1995-2000 | 1.39 | 0.32 | 0.88 , 2.2 | 1.16 | 0.23 | 0.79 , 1.71 |
| 2001-2005 | 1.46 | 0.34 | 0.93 , 2.3 | 1.09 | 0.21 | 0.74 , 1.59 |
| 2005 onwards | 1.52 | 0.35 | 0.97 , 2.4 | 1.05 | 0.2 | 0.72 , 1.54 |
| Use of anti HT in year prior to study entry | 0.86 | 0.03 | 0.81 , 0.93 | 0.98 | 0.02 | 0.95 , 1.01 |
| Use of statin in year prior to study entry | 0.86 | 0.02 | 0.82 , 0.9 | 1.2 | 0.02 | 1.16 , 1.24 |
| Use of NSAID in year prior to study entry | 1.08 | 0.03 | 1.03 , 1.13 | 1.16 | 0.02 | 1.12 , 1.2 |
| HbA1c at study entry (%) |  |  |  |  |  |  |
| HbA1c at study entry spl1 | 0.72 | 0.02 | 0.7 , 0.75 | 2.33 | 0.03 | 2.27 , 2.39 |
| HbA1c at study entry spl2 | 1.25 | 0.02 | 1.22 , 1.29 | 0.67 | 0.01 | 0.65 , 0.68 |
| HbA1c at study entry spl3 | 0.9 | 0.01 | 0.88 , 0.92 | 1.11 | 0.01 | 1.09 , 1.13 |
| HbA1c at study entry spl4 | 1.08 | 0.01 | 1.06 , 1.1 | 1.06 | 0.01 | 1.05 , 1.08 |
| BMI at study entry ( kg/m^2^) |  |  |  |  |  |  |
| BMI at study entry spl1 | 0.77 | 0.03 | 0.72 , 0.83 | 1.16 | 0.01 | 1.14 , 1.18 |
| BMI at study entry spl2 | 0.94 | 0.01 | 0.93 , 0.96 | 0.92 | 0.01 | 0.9 , 0.94 |
| BMI at study entry spl3 | 1.04 | 0.01 | 1.02 , 1.06 | 1.04 | 0.01 | 1.02 , 1.06 |
| History of CVD at study entry | 0.94 | 0.06 | 0.83 , 1.06 | 1.05 | 0.02 | 1.01 , 1.1 |
| History of CKD at study entry | 0.81 | 0.05 | 0.72 , 0.91 | 0.72 | 0.03 | 0.67 , 0.78 |
|  |  |  |  |  |  |  |
| TIME UPDATED COVARIATES |  |  |  |  |  |  |
| Use of anti HT in previous year | 1.08 | 0.03 | 1.03 , 1.13 |  |  |  |
| Use of statin in previous year | 1.65 | 0.04 | 1.57 , 1.73 |  |  |  |
| Use of NSAID in previous year | 1.22 | 0.04 | 1.14 , 1.31 |  |  |  |
| History of CVD | 1.02 | 0.06 | 0.91 , 1.15 |  |  |  |
| History of CKD | 0.9 | 0.04 | 0.83 , 0.98 |  |  |  |
| HbA1c in previous interval ( %) |  |  |  |  |  |  |
| Previous HbA1c spl1 | 5.48 | 0.09 | 5.31 , 5.66 |  |  |  |
| Previous HbA1c spl2 | 0.5 | 0.01 | 0.48 , 0.51 |  |  |  |
| Previous HbA1c spl3 | 1.48 | 0.02 | 1.43 , 1.52 |  |  |  |
| BMI in previous interval (per kg/m^2^ increase) | 1.07 | 0.01 | 1.06 , 1.08 |  |  |  |

Supplementary table 3: denominator and numerator model output for inverse probability of treatment weights using cubic spline covariate specifications.

Supplementary table 4: Parameter estimates from multinomial logistic regression model for probability of censoring, denominator model for weights (LEFT) and numerator model for stabilisation (RIGHT) Categorical covariate specification (primary analysis)

|  | DENOMINATOR MODEL | | | | | | | | | NUMERATOR MODEL | | | | | | | | |
| --- | --- | --- | --- | --- | --- | --- | --- | --- | --- | --- | --- | --- | --- | --- | --- | --- | --- | --- |
|  | **Censoring due initiation of other antidiabetic medication** | | | **Censoring due to death** | | | **Censoring due to transfer out** | | | **Censoring due initiation of other antidiabetic medication** | | | **Censoring due to death** | | | **Censoring due to transfer out** | | |
|  | OR | SE | 95% CI | OR | SE | 95% CI | OR | SE | 95% CI | OR | SE | 95% CI | OR | SE | 95% CI | OR | SE | 95% CI |
| BASELINE FIXED |  |  |  |  |  |  |  |  |  |  |  |  |  |  |  |  |  |  |
| Time (months) since study entry |  |  |  |  |  |  |  |  |  |  |  |  |  |  |  |  |  |  |
| Time since study entry (months) spl 1 | 0.91 | 0.03 | 0.86 , 0.96 | 1.42 | 0.06 | 1.26 , 1.62 | 1.06 | 0.05 | 0.96 , 1.17 | 0.56 | 0.03 | 0.53 , 0.59 | 1.57 | 0.06 | 1.38 , 1.77 | 1.00 | 0.05 | 0.9 , 1.09 |
| Time since study entry (months) spl 2 | 1 | 0 | 1 , 1.01 | 0.99 | 0.01 | 0.98 , 1.01 | 1 | 0.01 | 0.99 , 1.01 | 1.05 | 0 | 1.04 , 1.05 | 1 | 0.01 | 0.98 , 1.01 | 1.00 | 0.01 | 0.99 , 1.02 |
| Time (months) between diagnosis and study entry |  |  |  |  |  |  |  |  |  |  |  |  |  |  |  |  |  |  |
| Time between diagnosis and study entry spl 1 | 0.84 | 0.03 | 0.79 , 0.89 | 1.25 | 0.04 | 1.16 , 1.34 | 1.08 | 0.03 | 1.01 , 1.16 | 0.82 | 0.04 | 0.76 , 0.89 | 1.25 | 0.04 | 1.16 , 1.34 | 1.08 | 0.04 | 1.01 , 1.16 |
| Time between diagnosis and study entry spl 2 | 1.03 | 0.02 | 0.99 , 1.07 | 0.93 | 0.03 | 0.88 , 0.99 | 1.03 | 0.03 | 0.98 , 1.08 | 0.84 | 0.03 | 0.8 , 0.9 | 0.94 | 0.03 | 0.89 , 1 | 1.02 | 0.03 | 0.97 , 1.07 |
| Age at diagnosis (years) |  |  |  |  |  |  |  |  |  |  |  |  |  |  |  |  |  |  |
| 32-44 |  |  |  |  |  |  |  |  |  |  |  |  |  |  |  |  |  |  |
| 45-49 | 0.94 | 0.03 | 0.89 , 0.99 | 2.16 | 0.19 | 1.49 , 3.13 | 0.73 | 0.05 | 0.66 , 0.8 | 0.83 | 0.03 | 0.79 , 0.87 | 1.99 | 0.19 | 1.38 , 2.92 | 0.69 | 0.05 | 0.63 , 0.76 |
| 60-74 | 0.9 | 0.03 | 0.84 , 0.95 | 5.87 | 0.19 | 4.06 , 8.41 | 0.55 | 0.06 | 0.49 , 0.61 | 0.66 | 0.03 | 0.62 , 0.7 | 5.75 | 0.18 | 4.01 , 8.25 | 0.51 | 0.05 | 0.46 , 0.57 |
| 75-89 | 0.77 | 0.04 | 0.71 , 0.84 | 16.3 | 0.19 | 11.3 , 23.6 | 0.76 | 0.06 | 0.67 , 0.86 | 0.56 | 0.04 | 0.52 , 0.61 | 18.4 | 0.19 | 12.8 , 26.6 | 0.73 | 0.06 | 0.64 , 0.83 |
| Gender | 1.05 | 0.02 | 1.02 , 1.09 | 0.78 | 0.04 | 0.72 , 0.84 | 0.95 | 0.03 | 0.9 , 1.02 | 0.95 | 0.02 | 0.92 , 0.99 | 0.8 | 0.04 | 0.74 , 0.87 | 0.95 | 0.03 | 0.89 , 1.01 |
| Smoking Status |  |  |  |  |  |  |  |  |  |  |  |  |  |  |  |  |  |  |
| Non |  |  |  |  |  |  |  |  |  |  |  |  |  |  |  |  |  |  |
| Current | 1.05 | 0.02 | 1 , 1.09 | 2.2 | 0.05 | 1.99 , 2.46 | 1.06 | 0.04 | 0.98 , 1.15 | 1.11 | 0.02 | 1.05 , 1.15 | 2.27 | 0.05 | 2.05 , 2.53 | 1.07 | 0.04 | 0.98 , 1.16 |
| Ex | 1.06 | 0.02 | 1.02 , 1.09 | 1.4 | 0.04 | 1.28 , 1.52 | 0.99 | 0.03 | 0.92 , 1.06 | 1.04 | 0.02 | 1 , 1.07 | 1.39 | 0.04 | 1.27 , 1.51 | 0.98 | 0.03 | 0.91 , 1.05 |
| Alcohol consumption |  |  |  |  |  |  |  |  |  |  |  |  |  |  |  |  |  |  |
| non_drinker |  |  |  |  |  |  |  |  |  |  |  |  |  |  |  |  |  |  |
| ex-drinker | 1.07 | 0.04 | 0.98 , 1.16 | 1.28 | 0.08 | 1.08 , 1.51 | 0.93 | 0.07 | 0.8 , 1.07 | 1.02 | 0.04 | 0.94 , 1.12 | 1.3 | 0.08 | 1.11 , 1.54 | 0.92 | 0.07 | 0.8 , 1.07 |
| current drinker unknown | 1.15 | 0.06 | 1.02 , 1.3 | 1.58 | 0.11 | 1.26 , 1.97 | 1.4 | 0.1 | 1.17 , 1.7 | 1.17 | 0.07 | 1.04 , 1.34 | 1.6 | 0.11 | 1.27 , 1.99 | 1.43 | 0.1 | 1.2 , 1.73 |
| rare drinker <2u/d | 0.96 | 0.03 | 0.91 , 1.02 | 0.99 | 0.07 | 0.87 , 1.13 | 0.85 | 0.05 | 0.77 , 0.95 | 0.95 | 0.03 | 0.9 , 1.01 | 0.97 | 0.06 | 0.85 , 1.11 | 0.85 | 0.05 | 0.77 , 0.94 |
| moderate drinker 3-6u/d | 0.98 | 0.03 | 0.93 , 1.03 | 0.89 | 0.06 | 0.79 , 1 | 0.84 | 0.05 | 0.77 , 0.93 | 0.96 | 0.03 | 0.9 , 1.01 | 0.87 | 0.06 | 0.77 , 0.97 | 0.84 | 0.05 | 0.76 , 0.92 |
| excessive drinker >6u/d | 0.96 | 0.04 | 0.89 , 1.03 | 1.35 | 0.08 | 1.15 , 1.58 | 0.87 | 0.07 | 0.77 , 0.99 | 0.89 | 0.04 | 0.83 , 0.95 | 1.32 | 0.08 | 1.13 , 1.54 | 0.85 | 0.07 | 0.75 , 0.97 |
| Year of diabetes onset |  |  |  |  |  |  |  |  |  |  |  |  |  |  |  |  |  |  |
| 1990-1994 |  |  |  |  |  |  |  |  |  |  |  |  |  |  |  |  |  |  |
| 1995-2000 | 0.54 | 0.18 | 0.39 , 0.76 | 1.28 | 0.22 | 0.83 , 1.99 | 0.81 | 0.24 | 0.51 , 1.28 | 0.5 | 0.16 | 0.36 , 0.68 | 1.22 | 0.23 | 0.79 , 1.92 | 0.79 | 0.24 | 0.5 , 1.27 |
| 2001-2005 | 0.34 | 0.18 | 0.24 , 0.48 | 1.21 | 0.22 | 0.78 , 1.88 | 0.82 | 0.23 | 0.52 , 1.3 | 0.3 | 0.16 | 0.22 , 0.41 | 1.14 | 0.23 | 0.73 , 1.79 | 0.79 | 0.24 | 0.5 , 1.26 |
| 2005 onwards | 0.27 | 0.18 | 0.19 , 0.38 | 1.14 | 0.23 | 0.73 , 1.77 | 0.86 | 0.24 | 0.54 , 1.36 | 0.22 | 0.16 | 0.16 , 0.3 | 1.07 | 0.23 | 0.68 , 1.68 | 0.84 | 0.24 | 0.53 , 1.35 |
| Use of anti HT in year prior to study entry | 0.92 | 0.03 | 0.88 , 0.97 | 1.55 | 0.07 | 1.35 , 1.8 | 1.23 | 0.06 | 1.11 , 1.38 | 0.95 | 0.02 | 0.92 , 0.99 | 1.26 | 0.05 | 1.14 , 1.38 | 0.9 | 0.03 | 0.84 , 0.96 |
| Use of statin in year prior to study entry | 0.93 | 0.02 | 0.9 , 0.97 | 0.95 | 0.05 | 0.86 , 1.04 | 1.12 | 0.04 | 1.03 , 1.2 | 0.94 | 0.02 | 0.9 , 0.98 | 0.8 | 0.04 | 0.74 , 0.87 | 0.98 | 0.03 | 0.92 , 1.05 |
| Use of NSAID in year prior to study entry | 1.07 | 0.02 | 1.02 , 1.12 | 0.9 | 0.05 | 0.81 , 0.99 | 1.04 | 0.04 | 0.96 , 1.13 | 1.07 | 0.02 | 1.03 , 1.12 | 0.94 | 0.05 | 0.85 , 1.03 | 0.98 | 0.04 | 0.9 , 1.05 |
| HbA1c at study entry |  |  |  |  |  |  |  |  |  |  |  |  |  |  |  |  |  |  |
| <6% |  |  |  |  |  |  |  |  |  |  |  |  |  |  |  |  |  |  |
| 6% - 6.5% | 1.01 | 0.04 | 0.92 , 1.09 | 1.09 | 0.06 | 0.97 , 1.23 | 0.96 | 0.05 | 0.86 , 1.06 | 1.32 | 0.04 | 1.22 , 1.42 | 0.95 | 0.06 | 0.85 , 1.06 | 0.95 | 0.05 | 0.86 , 1.05 |
| 6.5%-7% | 1.11 | 0.04 | 1.02 , 1.2 | 1.15 | 0.07 | 1.01 , 1.31 | 1.16 | 0.06 | 1.04 , 1.3 | 1.72 | 0.04 | 1.6 , 1.84 | 0.99 | 0.06 | 0.88 , 1.11 | 1.15 | 0.05 | 1.04 , 1.27 |
| 7% - 8% | 1.12 | 0.04 | 1.03 , 1.21 | 1.3 | 0.07 | 1.13 , 1.49 | 1.14 | 0.06 | 1.02 , 1.28 | 2.23 | 0.04 | 2.08 , 2.41 | 1.14 | 0.06 | 1 , 1.28 | 1.17 | 0.05 | 1.06 , 1.3 |
| 8%-10% | 1.11 | 0.04 | 1.02 , 1.2 | 1.42 | 0.08 | 1.21 , 1.68 | 1.22 | 0.07 | 1.07 , 1.39 | 2.97 | 0.04 | 2.75 , 3.22 | 1.34 | 0.08 | 1.15 , 1.57 | 1.31 | 0.06 | 1.16 , 1.46 |
| >10% | 1.23 | 0.04 | 1.13 , 1.34 | 1.28 | 0.11 | 1.03 , 1.6 | 1.08 | 0.08 | 0.93 , 1.27 | 4.57 | 0.04 | 4.22 , 4.95 | 1.21 | 0.1 | 0.98 , 1.48 | 1.21 | 0.07 | 1.05 , 1.39 |
| BMI at study entry |  |  |  |  |  |  |  |  |  |  |  |  |  |  |  |  |  |  |
| <25 |  |  |  |  |  |  |  |  |  |  |  |  |  |  |  |  |  |  |
| 25-29 | 0.7 | 0.04 | 0.64 , 0.76 | 1.01 | 0.06 | 0.9 , 1.14 | 1.05 | 0.07 | 0.92 , 1.2 | 0.71 | 0.03 | 0.66 , 0.76 | 0.59 | 0.05 | 0.53 , 0.65 | 0.91 | 0.05 | 0.83 , 1.01 |
| 30-34 | 0.57 | 0.05 | 0.51 , 0.63 | 1.32 | 0.09 | 1.12 , 1.57 | 1.09 | 0.08 | 0.92 , 1.3 | 0.66 | 0.03 | 0.62 , 0.7 | 0.53 | 0.06 | 0.47 , 0.59 | 0.87 | 0.05 | 0.78 , 0.96 |
| 35+ | 0.5 | 0.06 | 0.44 , 0.57 | 1.75 | 0.12 | 1.38 , 2.23 | 1.04 | 0.11 | 0.84 , 1.28 | 0.69 | 0.03 | 0.64 , 0.74 | 0.61 | 0.06 | 0.53 , 0.68 | 0.83 | 0.06 | 0.74 , 0.92 |
| History of CVD at study entry | 0.99 | 0.05 | 0.9 , 1.09 | 0.74 | 0.07 | 0.64 , 0.85 | 0.83 | 0.09 | 0.69 , 0.98 | 1.16 | 0.03 | 1.09 , 1.22 | 1.77 | 0.04 | 1.63 , 1.93 | 1.08 | 0.05 | 0.99 , 1.19 |
| History of CKD at study entry | 1.15 | 0.06 | 1.03 , 1.28 | 1.12 | 0.08 | 0.96 , 1.31 | 0.9 | 0.1 | 0.76 , 1.09 | 1.32 | 0.05 | 1.2 , 1.45 | 1.52 | 0.07 | 1.32 , 1.73 | 0.91 | 0.08 | 0.78 , 1.06 |
| TIME UPDATED |  |  |  |  |  |  |  |  |  |  |  |  |  |  |  |  |  |  |
| Medication in previous interval | 2.44 | 0.02 | 2.34 , 2.56 | 0.89 | 0.05 | 0.81 , 0.98 | 0.88 | 0.04 | 0.82 , 0.94 | 4.26 | 0.02 | 4.06 , 4.44 | 0.83 | 0.05 | 0.76 , 0.91 | 0.85 | 0.04 | 0.79 , 0.91 |
| Use of anti HT in previous year | 1.07 | 0.02 | 1.03 , 1.13 | 1.12 | 0.05 | 1.01 , 1.25 | 0.86 | 0.05 | 0.79 , 0.94 |  |  |  |  |  |  |  |  |  |
| Use of statin in previous year | 1.12 | 0.02 | 1.06 , 1.16 | 0.7 | 0.05 | 0.64 , 0.77 | 0.79 | 0.04 | 0.73 , 0.86 |  |  |  |  |  |  |  |  |  |
| Use of NSAID in previous year | 1.16 | 0.03 | 1.11 , 1.22 | 0.68 | 0.07 | 0.59 , 0.79 | 0.66 | 0.06 | 0.58 , 0.73 |  |  |  |  |  |  |  |  |  |
| History of CVD | 1.14 | 0.05 | 1.04 , 1.25 | 2.64 | 0.07 | 2.29 , 3 | 1.34 | 0.08 | 1.14 , 1.57 |  |  |  |  |  |  |  |  |  |
| History of CKD | 1.31 | 0.04 | 1.22 , 1.42 | 1.49 | 0.05 | 1.34 , 1.65 | 1.02 | 0.06 | 0.9 , 1.15 |  |  |  |  |  |  |  |  |  |
| HbA1c in previous interval |  |  |  |  |  |  |  |  |  |  |  |  |  |  |  |  |  |  |
| <6% |  |  |  |  |  |  |  |  |  |  |  |  |  |  |  |  |  |  |
| 6% - 6.5% | 1.52 | 0.08 | 1.31 , 1.79 | 0.7 | 0.06 | 0.63 , 0.79 | 1 | 0.05 | 0.9 , 1.11 |  |  |  |  |  |  |  |  |  |
| 6.5%-7% | 2.75 | 0.07 | 2.36 , 3.16 | 0.66 | 0.06 | 0.59 , 0.75 | 0.93 | 0.05 | 0.84 , 1.04 |  |  |  |  |  |  |  |  |  |
| 7% - 8% | 11.7 | 0.07 | 10.2 , 13.5 | 0.7 | 0.07 | 0.61 , 0.79 | 1.02 | 0.06 | 0.91 , 1.15 |  |  |  |  |  |  |  |  |  |
| 8%-10% | 38.4 | 0.07 | 33.5 , 44.2 | 0.94 | 0.09 | 0.79 , 1.14 | 1.17 | 0.07 | 1.02 , 1.35 |  |  |  |  |  |  |  |  |  |
| >10% | 66.0 | 0.08 | 56.8 , 76.7 | 0.96 | 0.17 | 0.68 , 1.35 | 1.34 | 0.1 | 1.09 , 1.63 |  |  |  |  |  |  |  |  |  |
| Bmi in previous interval |  |  |  |  |  |  |  |  |  |  |  |  |  |  |  |  |  |  |
| <25 |  |  |  |  |  |  |  |  |  |  |  |  |  |  |  |  |  |  |
| 25-29 | 0.93 | 0.04 | 0.86 , 1.01 | 0.47 | 0.06 | 0.41 , 0.52 | 0.84 | 0.06 | 0.74 , 0.95 |  |  |  |  |  |  |  |  |  |
| 30-34 | 1 | 0.05 | 0.91 , 1.11 | 0.32 | 0.09 | 0.27 , 0.39 | 0.77 | 0.08 | 0.66 , 0.9 |  |  |  |  |  |  |  |  |  |
| 35+ | 1.2 | 0.06 | 1.06 , 1.34 | 0.31 | 0.13 | 0.24 , 0.4 | 0.8 | 0.1 | 0.66 , 0.98 |  |  |  |  |  |  |  |  |  |

supplementary table 5: Parameter estimates from multinomial logistic regression model for probability of censoring, denominator model for weights (LEFT) and numerator model for stabilisation (RIGHT) cubic spline covariate specification

|  | DENOMINATOR MODEL | | | | | | | | | NUMERATOR MODEL | | | | | | | | |
| --- | --- | --- | --- | --- | --- | --- | --- | --- | --- | --- | --- | --- | --- | --- | --- | --- | --- | --- |
|  | **Censoring due initiation of other antidiabetic medication** | | | **Censoring due to death** | | | **Censoring due to transfer out** | | | **Censoring due initiation of other antidiabetic medication** | | | **Censoring due to death** | | | **Censoring due to transfer out** | | |
|  | OR | SE | 95% CI | OR | SE | 95% CI | OR | SE | 95% CI | OR | SE | 95% CI | OR | SE | 95% CI | OR | SE | 95% CI |
| BASELINE FIXED |  |  |  |  |  |  |  |  |  |  |  |  |  |  |  |  |  |  |
| Time (months) since study entry |  |  |  |  |  |  |  |  |  |  |  |  |  |  |  |  |  |  |
| Time since study entry (months) spl 1 | 0.76 | 0.03 | 0.72 , 0.8 | 1.32 | 0.07 | 1.16 , 1.51 | 1.28 | 0.05 | 1.17 , 1.42 | 0.41 | 0.02 | 0.39 , 0.43 | 1.54 | 0.06 | 1.35 , 1.73 | 1.22 | 0.05 | 1.12 , 1.35 |
| Time since study entry (months) spl 2 | 1.27 | 0.03 | 1.2 , 1.34 | 1.01 | 0.07 | 0.88 , 1.16 | 0.8 | 0.05 | 0.73 , 0.9 | 2.41 | 0.03 | 2.27 , 2.53 | 0.99 | 0.07 | 0.86 , 1.14 | 0.84 | 0.05 | 0.76 , 0.93 |
| Time since study entry (months) spl 3 | 0.72 | 0.03 | 0.68 , 0.76 | 0.99 | 0.09 | 0.84 , 1.17 | 1.35 | 0.06 | 1.2 , 1.51 | 0.35 | 0.03 | 0.33 , 0.37 | 1.01 | 0.08 | 0.86 , 1.2 | 1.28 | 0.06 | 1.15 , 1.45 |
| Time since study entry (months) spl 4 | 1.14 | 0.02 | 1.11 , 1.17 | 1.03 | 0.04 | 0.96 , 1.11 | 0.9 | 0.03 | 0.86 , 0.96 | 1.38 | 0.02 | 1.34 , 1.42 | 1.02 | 0.04 | 0.95 , 1.09 | 0.91 | 0.03 | 0.87 , 0.97 |
| Time (months) between diagnosis and study entry |  |  |  |  |  |  |  |  |  |  |  |  |  |  |  |  |  |  |
| Time between diagnosis and study entry spl 1 | 0.85 | 0.03 | 0.8 , 0.9 | 1.27 | 0.04 | 1.19 , 1.36 | 1.08 | 0.03 | 1.02 , 1.16 | 0.81 | 0.04 | 0.75 , 0.88 | 1.26 | 0.04 | 1.17 , 1.35 | 1.08 | 0.04 | 1.02 , 1.16 |
| Time between diagnosis and study entry spl 2 | 1.04 | 0.02 | 1 , 1.08 | 0.92 | 0.03 | 0.87 , 0.99 | 1.03 | 0.03 | 0.98 , 1.08 | 0.84 | 0.03 | 0.79 , 0.89 | 0.95 | 0.03 | 0.89 , 1.01 | 1.02 | 0.03 | 0.97 , 1.08 |
| Age at diagnosis (years) |  |  |  |  |  |  |  |  |  |  |  |  |  |  |  |  |  |  |
| Age at diagnosis spl1 | 0.93 | 0.01 | 0.91 , 0.95 | 2.48 | 0.04 | 2.29 , 2.69 | 0.9 | 0.02 | 0.86 , 0.92 | 0.83 | 0.01 | 0.81 , 0.84 | 2.56 | 0.04 | 2.36 , 2.77 | 0.89 | 0.02 | 0.85 , 0.91 |
| Age at diagnosis spl2 | 0.97 | 0.01 | 0.95 , 0.99 | 1.08 | 0.04 | 1 , 1.16 | 1.19 | 0.01 | 1.15 , 1.22 | 1.01 | 0.01 | 0.99 , 1.02 | 1.15 | 0.04 | 1.07 , 1.23 | 1.21 | 0.01 | 1.17 , 1.23 |
| Age at diagnosis spl3 | 1.02 | 0.01 | 1.01 , 1.04 | 1.01 | 0.02 | 0.97 , 1.05 | 0.91 | 0.01 | 0.89 , 0.94 | 0.98 | 0.01 | 0.96 , 1 | 1.02 | 0.02 | 0.98 , 1.06 | 0.91 | 0.01 | 0.89 , 0.93 |
| Gender | 1.08 | 0.02 | 1.04 , 1.12 | 0.7 | 0.04 | 0.65 , 0.76 | 0.95 | 0.03 | 0.89 , 1.01 | 0.96 | 0.02 | 0.93 , 1 | 0.73 | 0.04 | 0.68 , 0.79 | 0.94 | 0.03 | 0.89 , 1.01 |
| Smoking Status |  |  |  |  |  |  |  |  |  |  |  |  |  |  |  |  |  |  |
| Non |  |  |  |  |  |  |  |  |  |  |  |  |  |  |  |  |  |  |
| Current | 1.04 | 0.02 | 0.99 , 1.08 | 2.32 | 0.05 | 2.08 , 2.59 | 1.06 | 0.04 | 0.97 , 1.15 | 1.09 | 0.02 | 1.05 , 1.14 | 2.41 | 0.05 | 2.18 , 2.66 | 1.06 | 0.04 | 0.98 , 1.16 |
| Ex | 1.06 | 0.02 | 1.02 , 1.11 | 1.39 | 0.04 | 1.27 , 1.52 | 0.99 | 0.03 | 0.93 , 1.06 | 1.05 | 0.02 | 1.01 , 1.08 | 1.38 | 0.04 | 1.26 , 1.51 | 0.99 | 0.03 | 0.92 , 1.06 |
| Alcohol consumption |  |  |  |  |  |  |  |  |  |  |  |  |  |  |  |  |  |  |
| non_drinker |  |  |  |  |  |  |  |  |  |  |  |  |  |  |  |  |  |  |
| ex-drinker | 1.06 | 0.04 | 0.98 , 1.16 | 1.32 | 0.08 | 1.12 , 1.55 | 0.94 | 0.07 | 0.81 , 1.08 | 1.02 | 0.04 | 0.94 , 1.12 | 1.35 | 0.08 | 1.14 , 1.58 | 0.93 | 0.07 | 0.81 , 1.07 |
| current drinker unknown | 1.16 | 0.06 | 1.02 , 1.31 | 1.55 | 0.12 | 1.23 , 1.95 | 1.42 | 0.1 | 1.17 , 1.72 | 1.19 | 0.06 | 1.04 , 1.34 | 1.62 | 0.11 | 1.3 , 2.01 | 1.45 | 0.1 | 1.2 , 1.75 |
| rare drinker <2u/d | 0.96 | 0.03 | 0.9 , 1.02 | 1.02 | 0.07 | 0.9 , 1.16 | 0.87 | 0.05 | 0.78 , 0.96 | 0.96 | 0.03 | 0.9 , 1.01 | 1.01 | 0.07 | 0.89 , 1.15 | 0.86 | 0.05 | 0.78 , 0.95 |
| moderate drinker 3-6u/d | 0.98 | 0.03 | 0.92 , 1.03 | 0.94 | 0.06 | 0.84 , 1.06 | 0.86 | 0.05 | 0.79 , 0.94 | 0.96 | 0.03 | 0.91 , 1.01 | 0.93 | 0.06 | 0.83 , 1.05 | 0.85 | 0.05 | 0.78 , 0.94 |
| excessive drinker >6u/d | 0.94 | 0.04 | 0.88 , 1.02 | 1.49 | 0.08 | 1.27 , 1.75 | 0.89 | 0.07 | 0.78 , 1.01 | 0.89 | 0.04 | 0.83 , 0.96 | 1.49 | 0.08 | 1.27 , 1.75 | 0.87 | 0.07 | 0.76 , 0.99 |
| Year of diabetes onset |  |  |  |  |  |  |  |  |  |  |  |  |  |  |  |  |  |  |
| 1990-1994 |  |  |  |  |  |  |  |  |  |  |  |  |  |  |  |  |  |  |
| 1995-2000 | 0.56 | 0.17 | 0.4 , 0.79 | 1.3 | 0.23 | 0.84 , 2.03 | 0.81 | 0.24 | 0.51 , 1.3 | 0.48 | 0.16 | 0.35 , 0.65 | 1.25 | 0.23 | 0.79 , 1.95 | 0.79 | 0.24 | 0.5 , 1.28 |
| 2001-2005 | 0.36 | 0.17 | 0.25 , 0.5 | 1.19 | 0.23 | 0.76 , 1.86 | 0.83 | 0.24 | 0.52 , 1.31 | 0.28 | 0.16 | 0.2 , 0.38 | 1.07 | 0.23 | 0.68 , 1.7 | 0.79 | 0.24 | 0.49 , 1.27 |
| 2005 onwards | 0.28 | 0.17 | 0.2 , 0.39 | 1.12 | 0.23 | 0.71 , 1.77 | 0.86 | 0.24 | 0.54 , 1.38 | 0.2 | 0.16 | 0.15 , 0.28 | 1.02 | 0.24 | 0.64 , 1.62 | 0.84 | 0.24 | 0.52 , 1.35 |
| Use of anti HT in year prior to study entry | 0.93 | 0.03 | 0.89 , 0.98 | 1.51 | 0.07 | 1.32 , 1.73 | 1.25 | 0.06 | 1.12 , 1.39 | 0.97 | 0.02 | 0.93 , 1.01 | 1.23 | 0.05 | 1.13 , 1.36 | 0.91 | 0.03 | 0.86 , 0.98 |
| Use of statin in year prior to study entry | 0.92 | 0.02 | 0.89 , 0.96 | 0.96 | 0.05 | 0.88 , 1.06 | 1.13 | 0.04 | 1.04 , 1.22 | 0.94 | 0.02 | 0.91 , 0.98 | 0.84 | 0.04 | 0.78 , 0.91 | 1.01 | 0.03 | 0.94 , 1.07 |
| Use of NSAID in year prior to study entry | 1.07 | 0.02 | 1.02 , 1.12 | 0.89 | 0.05 | 0.8 , 0.98 | 1.04 | 0.04 | 0.96 , 1.13 | 1.08 | 0.02 | 1.04 , 1.13 | 0.95 | 0.05 | 0.86 , 1.04 | 0.98 | 0.04 | 0.91 , 1.06 |
| HbA1c at study entry |  |  |  |  |  |  |  |  |  |  |  |  |  |  |  |  |  |  |
| HbA1c at study entry spl1 | 1.05 | 0.01 | 1.02 , 1.07 | 1.13 | 0.04 | 1.05 , 1.21 | 1.03 | 0.02 | 0.98 , 1.08 | 1.6 | 0.01 | 1.57 , 1.63 | 1.13 | 0.03 | 1.05 , 1.2 | 1.07 | 0.02 | 1.03 , 1.13 |
| HbA1c at study entry spl2 | 1 | 0.01 | 0.98 , 1.02 | 0.95 | 0.03 | 0.9 , 1.01 | 0.94 | 0.02 | 0.9 , 0.98 | 0.86 | 0.01 | 0.84 , 0.88 | 0.95 | 0.03 | 0.9 , 1 | 0.93 | 0.02 | 0.9 , 0.97 |
| HbA1c at study entry spl3 | 0.96 | 0.01 | 0.94 , 0.99 | 0.98 | 0.02 | 0.94 , 1.02 | 1.02 | 0.02 | 0.98 , 1.05 | 1 | 0.01 | 0.98 , 1.02 | 1.06 | 0.02 | 1.02 , 1.11 | 1.03 | 0.02 | 1 , 1.06 |
| HbA1c at study entry spl4 | 1.04 | 0.01 | 1.02 , 1.06 | 0.98 | 0.02 | 0.95 , 1.01 | 1.02 | 0.01 | 0.99 , 1.05 | 1.04 | 0.01 | 1.02 , 1.06 | 0.98 | 0.02 | 0.95 , 1.01 | 1.02 | 0.01 | 0.99 , 1.05 |
| BMI at study entry |  |  |  |  |  |  |  |  |  |  |  |  |  |  |  |  |  |  |
| BMI at study entry spl1 | 0.74 | 0.03 | 0.7 , 0.79 | 2.03 | 0.05 | 1.84 , 2.27 | 1.07 | 0.05 | 0.98 , 1.19 | 0.91 | 0.01 | 0.9 , 0.93 | 1.02 | 0.02 | 0.97 , 1.06 | 0.93 | 0.02 | 0.9 , 0.97 |
| BMI at study entry spl2 | 1.14 | 0.01 | 1.13 , 1.16 | 1.22 | 0.02 | 1.17 , 1.27 | 1.01 | 0.02 | 0.98 , 1.04 | 1.12 | 0.01 | 1.09 , 1.13 | 1.22 | 0.02 | 1.17 , 1.27 | 1.01 | 0.02 | 0.99 , 1.04 |
| BMI at study entry spl3 | 0.94 | 0.01 | 0.93 , 0.96 | 0.94 | 0.02 | 0.9 , 0.97 | 0.96 | 0.01 | 0.93 , 0.99 | 0.93 | 0.01 | 0.91 , 0.95 | 0.94 | 0.02 | 0.91 , 0.97 | 0.96 | 0.01 | 0.93 , 0.99 |
| History of CVD at study entry | 0.99 | 0.05 | 0.9 , 1.09 | 0.77 | 0.07 | 0.66 , 0.89 | 0.83 | 0.09 | 0.69 , 0.98 | 1.17 | 0.03 | 1.12 , 1.25 | 1.67 | 0.04 | 1.54 , 1.82 | 1.07 | 0.05 | 0.98 , 1.17 |
| History of CKD at study entry | 1.13 | 0.06 | 1.01 , 1.27 | 1.07 | 0.08 | 0.92 , 1.26 | 0.88 | 0.1 | 0.73 , 1.06 | 1.35 | 0.05 | 1.22 , 1.48 | 1.34 | 0.07 | 1.17 , 1.52 | 0.87 | 0.08 | 0.75 , 1.02 |
| TIME UPDATED |  |  |  |  |  |  |  |  |  |  |  |  |  |  |  |  |  |  |
| Medication in previous interval | 2.44 | 0.02 | 2.34 , 2.56 | 0.9 | 0.05 | 0.81 , 0.99 | 0.87 | 0.04 | 0.81 , 0.94 | 4.53 | 0.02 | 4.31 , 4.71 | 0.87 | 0.05 | 0.79 , 0.96 | 0.85 | 0.04 | 0.79 , 0.91 |
| Use of anti HT in previous year | 1.08 | 0.02 | 1.03 , 1.13 | 1.15 | 0.05 | 1.03 , 1.27 | 0.87 | 0.04 | 0.79 , 0.95 |  |  |  |  |  |  |  |  |  |
| Use of statin in previous year | 1.16 | 0.02 | 1.11 , 1.21 | 0.75 | 0.05 | 0.68 , 0.83 | 0.8 | 0.04 | 0.74 , 0.87 |  |  |  |  |  |  |  |  |  |
| Use of NSAID in previous year | 1.17 | 0.03 | 1.12 , 1.23 | 0.7 | 0.07 | 0.61 , 0.81 | 0.66 | 0.06 | 0.59 , 0.75 |  |  |  |  |  |  |  |  |  |
| History of CVD | 1.15 | 0.05 | 1.04 , 1.26 | 2.39 | 0.07 | 2.1 , 2.75 | 1.32 | 0.08 | 1.13 , 1.55 |  |  |  |  |  |  |  |  |  |
| History of CKD | 1.35 | 0.04 | 1.26 , 1.45 | 1.38 | 0.05 | 1.25 , 1.54 | 1.02 | 0.06 | 0.9 , 1.15 |  |  |  |  |  |  |  |  |  |
| HbA1c in previous interval |  |  |  |  |  |  |  |  |  |  |  |  |  |  |  |  |  |  |
| Previous HbA1c spl1 | 3.46 | 0.01 | 3.35 , 3.56 | 1.06 | 0.03 | 1 , 1.13 | 1.09 | 0.02 | 1.05 , 1.14 |  |  |  |  |  |  |  |  |  |
| Previous HbA1c spl2 | 0.68 | 0.02 | 0.66 , 0.71 | 1.08 | 0.03 | 1.03 , 1.14 | 1.02 | 0.02 | 0.99 , 1.06 |  |  |  |  |  |  |  |  |  |
| Previous HbA1c spl3 | 1.4 | 0.02 | 1.35 , 1.45 | 1.16 | 0.02 | 1.12 , 1.21 | 1.02 | 0.02 | 0.98 , 1.05 |  |  |  |  |  |  |  |  |  |
| BMI in previous interval | 1.03 | 0.01 | 1.02 , 1.04 | 0.88 | 0.01 | 0.86 , 0.9 | 0.98 | 0.01 | 0.96 , 0.99 |  |  |  |  |  |  |  |  |  |

| Length of time on metformin | Model 1 - basic baseline adjustment | Model 2 - Full baseline adjustment | Model 3 - Baseline and time updated adjustment | Model 4 – MSM with IPTW | Model 5 – MSM with IPTW and IPCW |
| --- | --- | --- | --- | --- | --- |
| 0-6 months | 0.83  (0.69 , 1.00) | 0.86  (0.71 , 1.04) | 0.86  (0.71 , 1.05) | 0.93  (0.68 , 1.27) | 0.91  (0.67 , 1.23) |
| 6-12 months | 0.97  (0.81 , 1.16) | 1.00  (0.83 , 1.21) | 0.99  (0.82 , 1.19) | 1.00  (0.75 , 1.35) | 0.87  (0.67 , 1.12) |
| 1-2 years | 0.89  (0.77 , 1.04) | 0.92  (0.79 , 1.08) | 0.91  (0.78 , 1.07) | 0.98  (0.76 , 1.26) | 0.96  (0.74 , 1.24) |
| 2-5 years | 0.95  (0.83 , 1.07) | 0.98  (0.86 , 1.12) | 0.97  (0.85 , 1.11) | 1.05  (0.84 , 1.31) | 1.04  (0.82 , 1.31) |
| 5-7 years | 0.95  (0.75 , 1.20) | 1.00  (0.78 , 1.27) | 0.99  (0.77 , 1.26) | 1.06  (0.73 , 1.53) | 1.09  (0.70 , 1.68) |
| >7 years | 0.85  (0.60 , 1.22) | 0.90  (0.62 , 1.29) | 0.88  (0.61 , 1.28) | 0.75  (0.41 , 1.36) | 1.00  (0.49 , 2.03) |

Supplementary table 8: HR and 95% CI for metformin vs no medication on risk of all cancers combined , by time since first prescription to metformin.

Estimates from three standard analysis methods (1-3) and one MSMs. with joint IPTW and IPCW (4).**Model 1** **– Minimal adjustment for confounding**: adjustment for age, gender, smoking status and alcohol status and year of onset of diabetes. **Model 2 – Full adjustment for baseline covariates**: Model 1 + baseline adjustment for: HbA1c, BMI, use of other medications in previous year (NSAIDS, statins, antihypertensive drugs), history of chronic kidney disease (CKD) and cardiovascular disease (CVD). **Model 3 – Full adjustment for baseline covariates with time-dependent covariates added:** Models 2 + adjustment for time updated HbA1c, BMI, and history of CVD, CKD and use of other medications in the past 12 months. **Model 4** – As Model 2, weighted using joint IPTW and IPCW (MSM with IPTW and IPCW). HRs approximated from a pooled logistic regressi

| Length of time on metformin | Model 1 - basic baseline adjustment | Model 2 - Full baseline adjustment | Model 3 - Baseline and time updated adjustment | Model 4 – MSM with IPTW | Model 5 – MSM with IPTW and IPCW |
| --- | --- | --- | --- | --- | --- |
| 0-6 months | 0.87  (0.7 , 1.06) | 0.87  (0.71 , 1.07) | 0.84  (0.68 , 1.05) | 0.99  (0.69 , 1.42) | 1.02  (0.73 , 1.44) |
| 6-12 months | 0.85  (0.68 , 1.06) | 0.86  (0.68 , 1.07) | 0.85  (0.68 , 1.06) | 0.86  (0.60 , 1.22) | 0.76  (0.57 , 1.03) |
| 1-2 years | 0.97  (0.82 , 1.15) | 0.98  (0.82 , 1.16) | 0.97  (0.81 , 1.16) | 1.03  (0.79 , 1.35) | 1.08  (0.81 , 1.43) |
| 2-5 years | 0.96  (0.83 , 1.11) | 0.97  (0.83 , 1.13) | 0.96  (0.82 , 1.12) | 1.11  (0.86 , 1.42) | 1.12  (0.86 , 1.45) |
| 5-7 years | 1.03  (0.80 , 1.34) | 1.03  (0.79 , 1.35) | 1.03  (0.79 , 1.35) | 1.12  (0.75 , 1.68) | 1.19  (0.74 , 1.93) |
| >7 years | 1.08  (0.74 , 1.58) | 1.08  (0.74 , 1.59) | 1.08  (0.73 , 1.59) | 1.01  (0.56 , 1.84) | 1.18  (0.56 , 2.49) |

Supplementary table 9: HR and 95% CI for metformin vs no medication on risk of all cancers combined (excluding NMSC) , by time since first prescription to metformin

Estimates from three standard analysis methods (1-3) and one MSMs. with joint IPTW and IPCW (4).**Model 1** **– Minimal adjustment for confounding**: adjustment for age, gender, smoking status and alcohol status and year of onset of diabetes. **Model 2 – Full adjustment for baseline covariates**: Model 1 + baseline adjustment for: HbA1c, BMI, use of other medications in previous year (NSAIDS, statins, antihypertensive drugs), history of chronic kidney disease (CKD) and cardiovascular disease (CVD). **Model 3 – Full adjustment for baseline covariates with time-dependent covariates added:** Models 2 + adjustment for time updated HbA1c, BMI, and history of CVD, CKD and use of other medications in the past 12 months. **Model 4** – As Model 2, weighted using joint IPTW and IPCW (MSM with IPTW and IPCW). HRs approximated from a pooled logistic regressi

Supplementary table 10: Hazard Ratio (HRR), 95% CI and p value for the effect of metformin vs no medication on risk of cancer in patients with newly diagnosed diabetes from 4 models with varying level of covariate adjustment using cubic spline parameterisations of baseline continuous covariates.

|  | All cancers (inc. NMSC) (2530 events) | | All cancers (excl. NMSC)  (2000 events) | | Breast Cancer  (241 events) | | Prostate Cancer  (266 events) | | Lung Cancer  (185 events) | | Pancreatic Cancer (50 events) | | Colorectal cancer  (226 events) | |
| --- | --- | --- | --- | --- | --- | --- | --- | --- | --- | --- | --- | --- | --- | --- |
|  | **HR** | **95% Confidence Interval** | **HR** | **95% Confidence Interval** | **HR** | **95% Confidence Interval** | **HR** | **95% Confidence Interval** | **HR** | **95% Confidence Interval** | **HR** | **95% Confidence Interval** | **HR** | **95% Confidence Interval** |
| Model 1 - basic baseline adjustment | 0.94 | (0.86 , 1.02) | 0.96 | (0.87 , 1.06) | 0.81 | (0.62 , 1.06) | 1.07 | (0.82 , 1.40) | 0.97 | (0.71 , 1.33) | 2.37 | (1.31 , 4.29) | 0.98 | (0.74, 1.30) |
| Model 2 - Full baseline adjustment | 0.97 | (0.88 , 1.07) | 0.97 | (0.87 , 1.08) | 0.84 | (0.62 , 1.14) | 1.13 | (0.84 , 1.53) | 0.99 | (0.71 , 1.38) | 2.06 | (0.99 , 4.30) | 0.88 | (0.64, 1.21) |
| Model 3 - Baseline and time updated adjustment | 0.96 | (0.87 , 1.06) | 0.96 | (0.85 , 1.08) | 0.89 | (0.65 , 1.22) | 1.16 | (0.85 , 1.57) | 1.01 | (0.72 , 1.40) | 1.61 | (0.80 , 3.23) | 0.80 | (0.59, 1.11) |
| Model 5 – MSM with IPTW and IPCW | 0.97 | (0.83 , 1.12) | 1.00 | (0.84 , 1.18) | 0.80 | (0.51 , 1.27) | 1.02 | (0.70 , 1.48) | 1.34 | (0.80 , 2.26) | 1.92 | (0.84 , 4.41) | 0.68 | (0.42, 1.14) |

Estimates from three standard analysis methods (1-3) and one MSMs. with joint IPTW and IPCW (4).**Model 1** **– Minimal adjustment for confounding**: adjustment for age, gender, smoking status and alcohol status and year of onset of diabetes. **Model 2 – Full adjustment for baseline covariates**: Model 1 + baseline adjustment for: HbA1c, BMI, use of other medications in previous year (NSAIDS, statins, antihypertensive drugs), history of chronic kidney disease (CKD) and cardiovascular disease (CVD). **Model 3 – Full adjustment for baseline covariates with time-dependent covariates added:** Models 2 + adjustment for time updated HbA1c, BMI, and history of CVD, CKD and use of other medications in the past 12 months. **Model 4** – As Model 2, weighted using joint IPTW and IPCW (MSM with IPTW and IPCW). HRs approximated from a pooled logistic regression


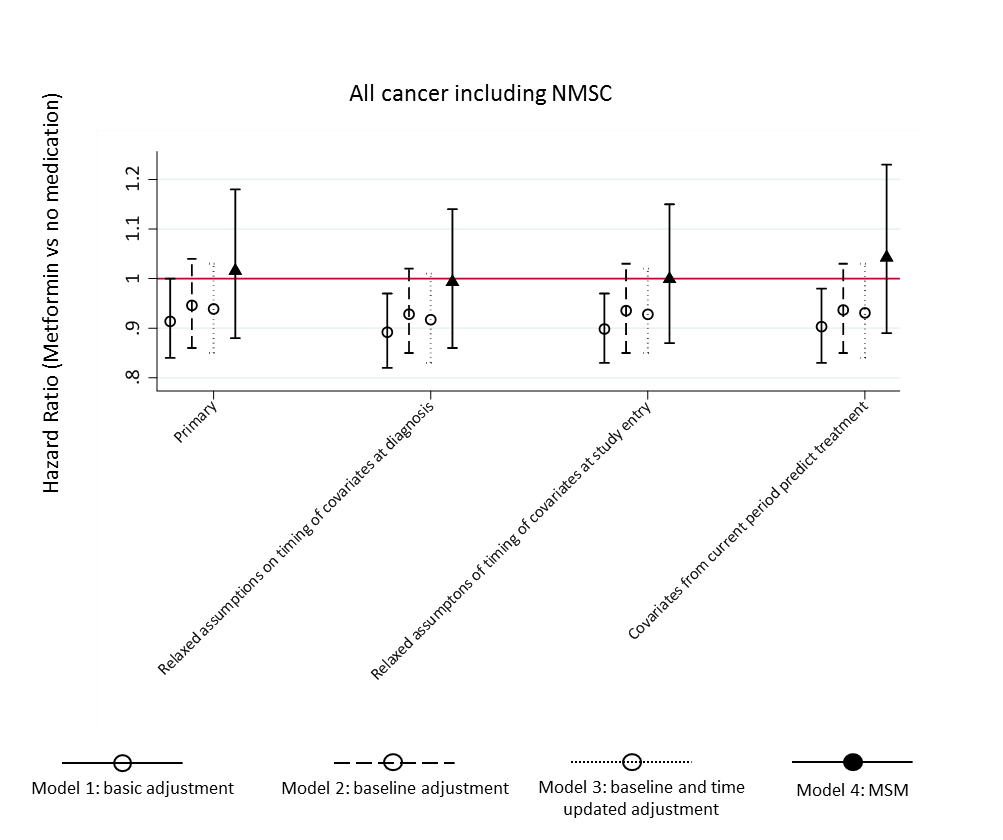


Supplementary figure 1: additional sensitivity analyses investigating impact of how data were set up to fit the MSMs.
