# Supplementary material for: Metformin use and risk of cancer in patients with type 2 diabetes: a cohort study of primary care records using inverse probability weighting of marginal structural models
Source: Int J Epidemiol. 2019 Feb 6;48(2):527–37. doi: 10.1093/ije/dyz005 (PMC6469299; doi:10.1093/ije/dyz005)
Supplement: Supplementary Data [file dyz005_supp.zip › dyz005-Suppl_data/dyz005_Supp_2.docx]

**Identification of patients with incident type 2 diabetes**

Pre-existing code lists for patients with diabetes have been developed for CPRD based on an algorithm originally developed for the UK Biobank data (1). These lists were developed in order to identify patients with prevalent type I and II diabetes.

Briefly, the UK Biobank algorithm extracts patients based on an initial diagnosis code list, ( referred to as primary diagnosis codes). The algorithm then aims to confirm diagnoses of diabetes by assessing the additional presence of

a) Oral antidiabetic medication

or

b) Codes relating to the care and management of diabetes within a patient’s medical history, termed “process of care” codes, such as “diabetic monitoring” and “diabetic annual review”.

Or

c) Presence of hyperglycaemia as indicated by HbA1c or glucose test results

The code “diabetic on diet only” is classified by BioBank as a process of care code, and not a diagnostic code. For the purposes of this study, “diabetic on diet only” was moved into the primary diagnosis code list, because initial data exploration suggested that this code was often used in CPRD to denote a diagnosis with no further diagnosis code present. Following the Biobank algorithm may have led to the exclusion of a lot of pre-medication follow up. All other process of care codes were used as in the original algorithm.

For some patients, information is also required on ethnicity, BMI, and age to judge whether it is likely that the patient has type 1 or type 2 diabetes. For this study, the dependence upon ethnicity was removed from the algorithm, and this part of the algorithm was based on BMI and age only. The reason for this was to improve the sample size by not excluding a large proportion of patients who did not have ethnicity recorded.

The flow chart below shows the algorithm used in this study and numbers included and excluded at each stage. .

Supplementary methods figure 1: Flow chart to show numbers identified as T2DM patients from August 2014 CPRD extract


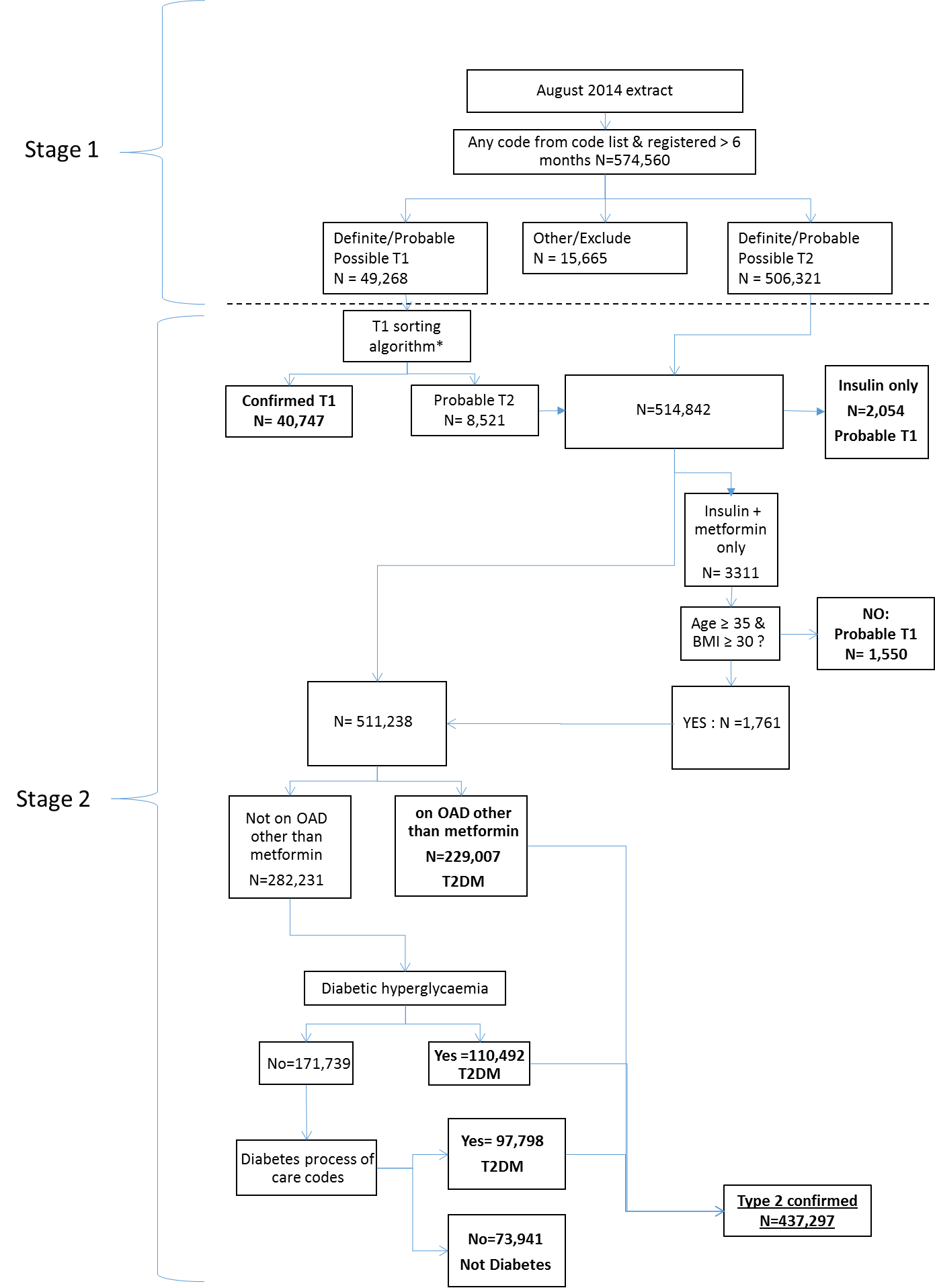


*see stage 2a below

**Stage 1:** In an initial step, primary diagnosis codes in a patient’s history were sorted to place the patient into their most likely category of the following: Definite Type 1, Definite Type 2, Probable Type 1, Probable Type 2, Possible Type 1, Possible Type 2, Vague codes (referring to codes that are unclear), Genetic diabetes, and Other Types (secondary, gestational, not diabetes, resolved diabetes). This was done as in (1).

**Stage 2 :** The second stages of the algorithm also required information on whether the patient had ever been prescribed insulin, metformin, or other OADs (see “identifying glucose lowering medications”), and also:

- The age of the patient at their date of onset.
- The BMI of the patients closest to their date of onset .
- Presence of hyperglycaemia (defined as HbA1c > 6.5% or fasting glucose >7.5 mmol/L) at the closest time to date of onset available.
- Process of care codes to indicate that the patient was receiving care for DM.

Two further sorting algorithms were then applied to the data.

**Stage 2a** : Firstly, those patients initially identified as definite, probable or possible type 1 were sorted and either kept as a type 1, or re-entered as possible type 2. In brief, patients entering this algorithm were immediately reclassified as possible type 2 if they had never been prescribed insulin; if they had been on an oral anti-diabetes drug other than metformin for more than 6 months; or if they were ever on insulin and metformin, or were overweight and over the age of 35 at the time of the first diagnosis code. All remaining patients were classified as having type 1 diabetes. The re classified patients were put back into the next stage of the algorithm.

**Stage 2b :** Secondly those initially sorted as definite, probable, possible type 2, vague, or patients coming back into the pool of possible patients from the type 1 algorithm were sorted by a second algorithm into type 2, probably type 1, and not diabetes, as shown in the flow chart.

## Defining onset, start of follow up, and final incident diabetes cohort

Once the initial cohort of 437,297 patients with T2DM was identified, a more specific date of diabetes onset was defined. This process was not part of the original Biobank algorithm, but was implemented to obtain a cohort of patients that could, with greater certainty, be followed up from time of diabetes diagnosis. In consideration of the order in which the algorithm priorities information, but also to maintain simplicity, the patient’s date of diabetes onset was taken to be the earliest of:

1. their earliest of a possible/probable/definite T2 diabetes code
2. the earliest process of care (POC) code
3. the earliest medication with either metformin or an OAD.

The data were further cleaned to ensure all patients within the final cohort had were of a reasonable age at time of onset. Patients aged < 30 or > 90 years at diabetes diagnosis were excluded, reducing the sample to 418,473.

For a patient to be considered an “incident” rather than a prevalent case, it was required that there were at least 12 months of history prior to their onset date with no diabetes related codes, reducing the sample size to 219,346. Since multiple codes define the inclusion of a patient into the cohort, it was important to ensure that a patient did not begin follow up until the time when the minimum number of codes that defined inclusion had occurred. Alongside the diagnosis code, the three points in the algorithm that defined inclusion were either a prescription for an oral anti-diabetes drug other than metformin, a test indicating hyperglycaemia, or a process of care code. For simplicity (since 95% of patients with a test indicating hyperglycaemia also had a process of care code which would have ensured their inclusion regardless of the test result), the date of cohort eligibility was defined as the latest of

a) the first T2 diabetes code, and

b) the first process of care code OR first medication with an OAD other than metformin.

The follow up start date and the date of onset were required to be within 30 days of each other to ensure the patient was still an incident case of diabetes when entering the cohort (reducing to n= 117,775.). Finally, all patients were required to have had no history of cancer at the time of diabetes diagnosis (final n=98080).

## Identifying glucose lowering medications

Medications were identified from the complete product code list using British National Formulary (BNF) codes (2). All codes starting 060101, 060102, and excluding 06010103 were extracted – relating to subchapters Insulin, antidiabetic drugs, and excluding Hypodermic equipment respectively. Partial string matches were then used on the “drugsubstance” field to identify the different anti diabetic drug classes. These codes were merged with the patient history to obtain longitudinal prescription histories of anti-diabetic medications.

The exact code used to define the medication categories is detailed below

gen diabetestreat = 0

replace diabetestreat=1 if strmatch(bnfcode, "060101*") ///

| strmatch(bnfcode, "060102*")

replace diabetestreat=0 if strmatch(bnfcode, "06010103*")

gen nodrug = 0

replace nodrug=1 if strmatch(drugsubstance ,"*None known*")

gen diab_therapy=.

replace productname = lower(productname)

replace bnfchapter = lower(bnfchapter)

replace diab_therapy=1 if regexm(bnfcode, "60101") | regexm(productname, "insulin") | regexm(productname, "Insulin")

replace diab_therapy=2 if regexm(bnfcode, "60102")

replace diab_therapy=3 if ( strmatch(drugsubstance, "*metformin*") | regexm(productname, "Metformin") ) & strmatch(drugsubstance, "*/*")!=1

lab define diab_therapy 1 insulin 2 oad 3 metformin

lab val diab_therapy diab_therapy

Other OADs (diab_therapy=2), were then further classified using partial string matches and bnfcodes as detailed below, and checked manually.

**Sulfonylureas**: bnfcode 6.1.2.1 (plus partial string match for drug substance starting with “Gli” or ending in “mide”)

**Glitazones**: string search for drug substance ending in “zone”

**Glinides**: string search for drug substance ending in “glinide”

**DPP4:** string search for drug sunstance ending in “gliptin”

**GLPs**: string search for drug substance ending in “tide” excluding pramlintide.

# **Covariates**

## Time invariant confounders

**Sex and Age**

Sex was obtained directly from the patients record.

Age at diabetes diagnosis was calculated as year of diagnosis minus year of birth (available in the patients record).

**Smoking and Alcohol**

For simplicity, smoking and alcohol consumption were considered as time invariant. Smoking status is a specific entry field in the CPRD (entity type 4). The raw entry in CPRD classifies the smoking status into not entered, smoker, non-smoker and ex-smoker. All available entries were extracted for a patient. For multiple entries on the same day, if any “not entered” appeared alongside another code, then the not entered code was deleted. Any other discrepancies on the same day were changed to missing. Differences in coding were then examined longitudinally prior to the date of diabetes diagnosis. The closest entry prior to the date of diabetes diagnosis was examined. If this was current or ex-smoker, it was kept as such. If it was non-smoker, then it was only kept as non-smoker if all entries prior to this entry (if any) were also non-smoker or unknown. If any previous entry suggested the patient was a current or ex-smoker, they were changed to ex-smoker. This value was then taken as the smoking status of the patient at the time of diabetes diagnosis.

Alcohol consumption was determined via use of both read codes (see codelists) and information on reported daily and weekly alcohol consumption obtained from the additional information file (entity type 5).

The pre-existing code list had been manually sorted into 7 simple categories of “non-drinker”, “ex drinker”, “rare drinker”, “current drinker”, “excessive drinker”, and “drinker but amount not specified” and “missing”.

If available, the more detailed information on daily and weekly units were also used. The threshold of units per day or week to classify rare, moderate and excessive drinking was applied as follows:. Based on weekly information, rare was <1 unit per week for males and females, current was 1-21 (inclusive) units per week for males and 1-14 (inclusive) units per week for females. Excessive was more than 21 units per week for males and >14 units per week for females.

Daily information was classified such that <2 units per day was rare, 3-6 units per day as moderate, and >6 units per day as excessive, for both males and females.

All three classifications were then considered together to calculate the most appropriate category for each patient on each date. Discrepancies between clinical, weekly and daily info were dealt with a simple rule. As with smoking, a non-missing entry took priority over a missing entry. If there were two differing entries on the same day, the value taken was the worst case (higher quantity).

The closest record to the time of diabetes onset (but before) was then identified and any prior entries that were the same alcohol category were deleted for simplicity. A similar process to the smoking variable was then employed. If the closest code to diagnosis was non-drinker, but ex-drinker appeared previously, they were considered an ex-drinker. If either of current or rare drinker appeared previously, they were considered a rare drinker.

## Time-dependent confounders

**HbA1c and BMI**

HbA1c records, consisting of the value recorded, unit of measurement and date of record, were extracted (entity type 275). Results were only kept if they were entered as %, mmol/L, iu/L and mmol/mol. (this accounted for 80% of all entries, with a further 19% being labelled as “unit not entered”). All results were then converted to %. After conversion, readings of <2% and >20 % were considered implausible and deleted. Duplicates on the same day were then identified. Any result originally in % format was given priority. Multiple readings in % were then compared and removed if the difference was > 2%. Otherwise a mean was used. For patients entering the study at time of diabetes diagnosis, the HbA1c was required to have been within 6 months otherwise it was considered missing. Values below and above the 1^st^ and 99^th^ percentiles of the distribution were truncated by setting them to the 1^st^ and 99^th^ percentile values.

Height and weight were extracted (entity types 13 and 14), and merged with the patient records to obtain the dates of the entries. Height was cleaned by first searching for values >100, suggesting height had been entered in cm. These were converted to m. After this, entries suggesting implausible values ( >2.3 m (7.5 feet) and <1.3 m (4 feet)) were set to missing. Multiple height entries on the same day were replaced with the mean if the difference was less than 5 cm, or both created missing. Weight was then cleaned in a similar way, with implausible values removed (<4 stone (25kg) or > 40 stone (255kg)). Multiple entries on the same day were replaced with means, or removed if the differences between the two results differed by 2kg or more. BMI was then calculated using the cleaned height and weight measures, and the set of longitudinal measures for each patient saved. The cut of off 6 months prior to time of diagnosis for a valid measure was also applied for BMI. As with HbA1c, values were truncated at the 1^st^ and 99^th^ percentiles.

**Comorbidities**

In order to create a simple measure of whether a patient may have decreased kidney function, the quality outcome framework (QOF) (3) preferred and accepted codes for stages of chronic kidney disease were used as a set of Read codes to identify patients with CKD. A patient was considered to have no CKD until the time at which the first code suggesting CKD appeared, and at this point, presence of CKD was updated to reflect this. A separate variable was also created to represent the presence or not of CKD at baseline. The variable combined stages 3, 4 and 5 together. Initial investigations suggested that the number of patients initiating metformin with stage 4 or 5 CKD was too low to allow a finer categorisation than this.

Presence of CVD was defined using the QOF preferred and accepted codes (3) for ischaemic heart disease, acute myocardial infarction, all types of stroke and transient ischemic attack (TIA). The date of first record of any CVD was recorded and a patient’s data updated from the time of this code onwards to reflect presence of CVD. As with CKD, a separate record to identify if it was already present at study entry as also created.

**Concomitant medications**

Use of these three medications was established by searching the prescription data for the relevant BNF codes.For each patient, longitudinal data were collected consisting of the dates of prescriptions for each medication type for their whole history. This was used to calculate variables indicating use in the year prior to study entry. Equivalent time dependent variables for use in the previous year for each interval of follow up were also created.

The BNF codes used were as follows:

Statins: 02120400.

Anti-hypertensive medications: any thiazide diuretic (02020100) (plus any combinations including this), any calcium channel blocker (02060200), Angiotensin-converting enzyme (ACE) inhibitors (02050501), Angiotensin-II receptor antagonists (02050502) and Beta Blockers (02040000).

Non-steroidal anti-inflammatory medications (NSAIDs):100101

# **Assigning variables to intervals**

For intervals in which BMI or HbA1c records did not occur, last one carried forward (LOCF) was used. This assumes that this most recent value is the most representative of the true value during this time. Due to the likely lag between measurement and recording of HbA1c, it was decided that an HbA1c entered into a patient’s record would be considered to have actually occurred 7 days before. The same was done for CKD diagnoses, as, if not already present in the patient’s history by the time of diabetes diagnosis, these are likely to be in response to an eGFR or creatinine test, or a hospital diagnoses that may take time to reach the GP. All other covariates were considered to have occurred on the day indicated by the event date in the record.

# **Fitting MSMs with IPTW**

Follow up for each subject was split into discrete 1 month time intervals $t = 1\ldots T$. In each interval, information was required on treatment, covariates, outcome and a censoring indicator $C(t)$.

The models were fitted in two stages. Firstly, each individual’s probability of having their own treatment history was calculated; and used to calculate the IPTW (the treatment models). In the second stage, the treatment-outcome association was estimated in a regression model weighted using the IPTW (the outcome model).

### Calculating the IPTW

We define $A\left( t \right) \mathrm{and} \underline{A}\left( t^{-} \right)$ as the value of treatment in interval *t* and the full treatment history to interval *t-1* respectively; $L\left( t \right),$ and $\underline{L}\left( t^{-} \right)$as the analogous variables for a set of time-dependent covariates L; $V$ to be the vector of baseline covariates (where V is a subset of L); and $C\left( t \right)$ be an indicator for whether a subject is uncensored up to the end of interval *t* (with 0 indicating uncensored, and 1 indicating censored). We calculated stabilised inverse probability of treatment weights (4), as:

$SW\left( t \right)= \prod_{k=1}^{t} \frac{P[A(k)| C\left( k \right)=0,\underline{A}\left( k^{-} \right),V ]}{P[A\left( k \right)|C\left( k \right)=0,\underline{A}\left( k^{-} \right) ,\underline{L}\left( k^{-} \right), V]}$ (5)

Since we assumed that once treated the subject remained treated, we estimated these two probabilities for each time point by fitting two pooled logistic regression models: one for the numerator and one for the denominator. These models included data from all time intervals up to and including the first interval in which A(t)=1, and we set $P\left( A\left( t \right) \right)= 1 if A\left( t-1 \right)=1.$ For a survival outcome, the probability of treatment initiation must also be conditional on the outcome not occuring in interval $t$. In practical terms, this means fitting the weighting model in those who have not had the event up to the end of interval $t$.

Informally, the denominator of the stabilised weight is an individual’s probability of receiving their treatment in interval t, conditional on past treatment history $\underline{A}\left( k^{-} \right)$ , covariate history$\underline{L}\left( k^{-} \right)$., and baseline covariates $V$ (where $V$ may be a subset of $L$). The numerator is an individual’s probability of receiving their treatment in interval t, conditional on past treatment history $\underline{A}\left( k^{-} \right)$ and baseline covariates $V$ only. In the simple case, this translates to emulating a population where sequential treatment in patients still off treatment is random conditional on baseline confounders and treatment history.

### Censoring

Loss to follow up was accounted for via the use of inverse probability of censoring weights (IPCW)(6).

Then the IPCW at time $t$, denoted $CW(t)$, was defined as :

$CW\left( t \right)= \prod_{k=1}^{t} \frac{P[C(k) |\underline{A}\left( k^{-} \right) ,C\left( k-1 \right)=0,V]}{P[C\left( k \right)|\underline{A}\left( k^{-} \right) , C\left( k-1 \right)=0,, \underline{L}\left( k^{-} \right), V]}$ (5)

$SW(t)$ and $CW(t)$ can then be multiplied together, in order to obtain a joint inverse probabiltiy weight. This can be thought of as the inverse of the joint probability of observed treatment and remaining uncensored (4). The resulting weighted population would be interpreted as a population in which there is random treatment allocation with respect to risk of outcome, and no loss to follow up, conditional on baseline covariates and treatment history.

### Fitting the MSM

Stata is limited in that time updated weights cannot be applied to a time-varying Cox model. Therefore, as described in (5), we approximated this by a pooled logistic regression (7).

Formally, for an event $Y$, we defined the outcome at each time point $t = 1\ldots T$, to be $Y(t)$.$Y(t) =0$ for all intervals until the interval in which the event occurs, at which point $Y(t) = 1$. We modelled the pooled logistic regression as:

$logit\left[ P\left( Y\left( t \right)=1 \right|Y\left( t-1 \right)=0,C\left( t \right)=0, \underline{A}(t^{-}), V \right) ]= \alpha(t)+ \beta\underline{A}\left( t^{-} \right) + \boldsymbol{\gamma}V$ (5)

Note that we estimate $Y(t)$ to be conditional on $C(t) = 0$. That is, we only evaluated the outcome in those remaining uncensored to the end of interval $t$.

Since the risk of the outcome was evaluated with respect to **previous** treatment history, i.e. $Y(t)$ depends on$\underline{A}(t^{-})$, this implies that the weight for time $t$ in the model for the MSM must then relate to the probability of $A(t-1)$ and $C\left( t \right).$More formally, by the above notation, the combined weight for interval $t$ was defined as

$$CW(t) x SW(t-1)$$

with $SW(-1)$ set to 1.

Because the stabilisation of the weight means that treatment initiation is balanced conditional on baseline covariates, all variables $V$ included in the numerator of the stabilised weight were included in the outcome model (8). This model must also account for the dependence between observations from the same subject which are introduced by the weighting process, and therefore the variance was estimated by use of a robust variance estimator as described by Hernan, Brumback and Robins (8).

## Including patients treated from study entry

In order to ensure treatment has not already affected covariates, patients were required to be treatment free at the study entry. For patients that entered the study at the time of diabetes diagnosis, the decision was made to allow patients who initiated metformin within the 30 day period that defined inclusion into the cohort to be included, as long as all baseline covariates were measured strictly before the date of treatment initiation. These patients would always have a probability of treatment of 1, and so had a constant weight of 1 in the outcome model. These patients did not contribute to the models for the IPTW. Difference in baseline covariates between these patients and those who are untreated at baseline would be adjusted for since all baseline covariates are included in the outcome model.

# **Sensitivity analyses**

To examine how various modelling decisions may have affected the results of the MSMs, the treatment and censoring weights were re-calculated and the outcome models re-run for the scenarios (listed below) as sensitivity analyses.

## Dividing time into 3-month instead of 1-month intervals

Initial analysis suggested that a patient’s HbA1c and BMI were updated on average just over every 6 months, therefore use of 1 month intervals may have resulted in lots of information carried forward for these variables. This sensitivity analysis was conducted to investigate whether the results could be altered if larger intervals were used.

## Different lags on the cancer diagnosis

In the primary analysis, the date of cancer diagnosis was moved forward in time by 6 months to allow for un-diagnosed cancer. This was because developing cancer may affect diabetes symptoms and therefore affect treatment decisions. To test the sensitivity of the models to this assumption (i.e. to assess the potential impact of reverse causation on results), two sensitivity analyses were performed, one where the cancer diagnosis date was shifted forward by 12 months, and one where it was not changed at all.

## Relaxing assumptions of temporality between covariate measurement and treatment (2 analyses)

As explained in the main text and supplemental methods, we required treatment initiation to occur at or after study entry. In the primary analysis, this meant that patients who initiated treatment in the same interval in which they obtained complete data on HbA1c and BMI were excluded. This group of patients could be divided into two distinct groups: Firstly, patients initiating at time of diagnosis whose baseline covariates were measured after treatment initiation (as explained in “including patients treated from study entry”); and secondly, patients who initiated treatment post diabetes diagnosis but in the same interval in which they obtained complete data on BMI and HbA1c (as explained in the main text). These two sensitivity analyses looked at the impact of including these two groups of patients in the analyses.

## Using covariates in current interval to predict treatment in weight calculation

By using covariates from the previous interval to predict treatment initiation and censoring, we may not have correctly controlled for confounding at the time the treatment decision was actually made. This is because for many covariates, the true value may be measured at a GP consultation and treatment initiated (or not) immediately in response to this. By using covariates from the same interval to predict treatment initiation, it was possible to investigate whether the potential to be missing the true value that represents outcome risk and drives the treatment decisions may have influenced the estimated relative risks.

## Fitting different treatment models for each calendar period

In the overall model fitting process, potential interactions between covariates were not examined. Due to changing guidelines, there was some concern that calendar time may have been an important effect modifier for the effect of covariates on probability of treatment. For example, in the 1990s, those patients who were not overweight were more likely to be prescribed a sulfonylurea than metformin, however more recent guidelines indicate that metformin should be used in preference. Therefore, the effect of BMI on probability of treatment may be modified by calendar time. To investigate the impact this may have had on the results, IPTW and IPCW were estimated separately for patients diagnosed in different calendar periods. Due to small numbers for some time intervals, this analysis was restricted to a smaller range of calendar periods, including only patients diagnosed from the year 2000 onwards.

# **All codelists are available on the LSHTM data compass**

# **References**

1. Eastwood SV, Mathur R, Atkinson M, et al. Algorithms for the capture and adjudication of prevalent and incident diabetes in UK Biobank. PLoS ONE. 2016;11(9):e0162388.

2. British National Formulary. BNF volume 64: BMJ Group/Pharmaceutical Press; 2012.

3. NHS Digital. QOF business rules v30.0 2015 [cited 2015 January 18th]. Available from: <http://content.digital.nhs.uk/qofbrv30>.

4. Robins JM, Hernán MA, Brumback B. Marginal structural models and causal inference in epidemiology. Epidemiology. 2000;11(5):550-60.

5. Fewell Z, Hernan MA, Wolfe F, Tilling K, Choi HK, Sterne JAC. Controlling for time-dependent confounding using marginal structural models. The Stata Journal. 2004;4(4):402-20.

6. Robins JM, Finkelstein DM. Correcting for noncompliance and dependent censoring in an AIDS clinical trial with inverse probability of censoring weighted (IPCW) log-rank tests. Biometrics. 2000;56(3):779-88.

7. D'Agostino RB, Lee ML, Belanger AJ, Cupples LA, Anderson K, Kannel WB. Relation of pooled logistic regression to time dependent Cox regression analysis: the Framingham Heart Study. Statistics in Medicine. 1990;9(12):1501-15.

8. Hernán MA, Brumback B, Robins JM. Marginal structural models to estimate the causal effect of zidovudine on the survival of HIV-positive men. Epidemiology. 2000;11(5):561-70.
